# Nanopore decoding with speed and versatility for data storage

**DOI:** 10.1093/bioinformatics/btaf006

**Published:** 2025-01-08

**Authors:** Kevin D Volkel, Paul W Hook, Albert Keung, Winston Timp, James M Tuck

**Affiliations:** Department of Electrical and Computer Engineering, North Carolina State University, Raleigh, NC, 27606, United States; Department of Biomedical Engineering, Johns Hopkins University, Baltimore, MD, 21218, United States; Department of Chemical and Biomolecular Engineering, North Carolina State University, Raleigh, NC, 27695, United States; Department of Biomedical Engineering, Johns Hopkins University, Baltimore, MD, 21218, United States; Department of Electrical and Computer Engineering, North Carolina State University, Raleigh, NC, 27606, United States

## Abstract

**Motivation:**

As nanopore technology reaches ever higher throughput and accuracy, it becomes an increasingly viable candidate for reading out DNA data storage. Nanopore sequencing offers considerable flexibility by allowing long reads, real-time signal analysis, and the ability to read both DNA and RNA. We need flexible and efficient designs that match nanopore’s capabilities, but relatively few designs have been explored and many have significant inefficiency in read density, error rate, or compute time. To address these problems, we designed a new single-read per-strand decoder that achieves low byte error rates, offers high throughput, scales to long reads, and works well for both DNA and RNA molecules. We achieve these results through a novel soft decoding algorithm that can be effectively parallelized on a GPU. Our faster decoder allows us to study a wider range of system designs.

**Results:**

We demonstrate our approach on HEDGES, a state-of-the-art DNA-constrained convolutional code. We implement one hard decoder that runs serially and two soft decoders that run on GPUs. Our evaluation for each decoder is applied to the same population of nanopore reads collected from a synthesized library of strands. These same strands are synthesized with a T7 promoter to enable RNA transcription and decoding. Our results show that the hard decoder has a byte error rate over 25%, while the prior state of the art soft decoder can achieve error rates of 2.25%. However, that design also suffers a low throughput of 183 s/read. Our new Alignment Matrix Trellis soft decoder improves throughput by 257× with the trade-off of a higher byte error rate of 3.52% compared to the state of the art. Furthermore, we use the faster speed of our algorithm to explore more design options. We show that read densities of 0.33 bits/base can be achieved, which is 4× larger than prior MSA-based decoders. We also compare RNA to DNA, and find that RNA has 85% as many error-free reads when compared to DNA.

**Availability and implementation:**

Source code for our soft decoder and data used to generate figures is available publicly in the Github repository https://github.com/dna-storage/hedges-soft-decoder (10.5281/zenodo.11454877). All raw FAST5/FASTQ data are available at 10.5281/zenodo.11985454 and 10.5281/zenodo.12014515.

## 1 Introduction

DNA has emerged as a viable data storage medium in recent years, with advancements focused on reducing synthesis costs (Nguyen *et al.* 2021), improving encoding densities (Choi *et al.* 2019), and selectively retrieving information from DNA libraries (Organick *et al.* 2018). While many of the early works assumed high-throughput sequencing technologies, the sequencing technology landscape has seen major changes due to the continual advancements in yield and accuracy in nanopore sequencing devices and their basecalling algorithms (Wang *et al.* 2021, Pagès-Gallego and de Ridder 2023). The ability to reach yields of 100 Gb per flow cell make nanopore sequencing a competitive option for large-scale molecular storage systems in addition to portable ones (Yazdi *et al.* 2017). Furthermore, nanopore sequencing enables long read sequencing, real-time signal analysis (Loose *et al.* 2016, Kovaka *et al.* 2021), and can directly interrogate other biopolymers such as RNA. Hence, nanopore sequencing has the potential to support a wide range of interesting storage system architectures, but few of these options have been deeply explored.

A current bottleneck for nanopore-based DNA storage systems is their high cost of decoding. Most studies rely on *post hoc* multi sequence alignment (MSA) and clustering analyses as a critical decoding step to merge information across multiple reads of the same encoded molecule (Organick *et al.* 2018, Antkowiak *et al.* 2020). While some works may be able to write information at a density of 1.33 bits/base (Chen *et al.* 2021), read density can be over an order of magnitude lower (0.079 bits/base) due to reading each base 16.8× times on average in order to build a consensus read that will correctly decode (Supplementary Fig. S1). In the context of a storage system, this implies that the computational infrastructure supporting the decoding process and the sequencing material costs will be 16.8× larger than if each originally encoded strand was read once.

Convolutional codes (Chandak *et al.* 2020, Press *et al.* 2020) have shown great promise for single-read approaches that can extract the information payload of a sequence from a single read. HEDGES (Press *et al.* 2020) is a convolutional code that is tolerant of insertions and deletions and has been designed specifically for *single read*, but it has only been evaluated for illumina-based sequencing platforms, which have an order of magnitude lower error rate (0.1% in Organick *et al.* 2018, Tomek *et al.* 2019) than to nanopore sequencing. HEDGES tolerates errors by systematically and serially guessing the location of errors, which can significantly increase compute time under the higher error rates of nanopore. Chandak *et al.* have shown that a *soft decoding* technique that directly integrates the base probabilities output by nanopore basecallers can substantially lower read costs and byte error rates. However, the decode throughput is low, 183 s/read on average based on our benchmarking measurements.

Building on the success of prior convolutional codes and basecaller integration (Chandak *et al.* 2020), we explored two trellis soft decoders for the HEDGES encoding that can run in parallel on a GPU which aim to determine the message with the highest likelihood based on two distinct approaches to calculating the likelihood based on nanopore machine learning model outputs and solve for the most likely decoding. First, we integrate Chandak *et al.*’s trellis with the constrained encoding of HEDGES and parallelize it to run on a GPU. We use this as our baseline comparison. Second, we developed a new decoding algorithm that leverages a dynamic programming approach to compute trellis state probabilities in a way that can efficiently utilize the GPU’s parallelism and memory architecture.

In this work, we perform a systematic comparison between hard decoding and each soft decoder. First, we use HEDGES on state-of-the-art nanopore basecallers from Oxford Nanopore Technologies (ONT) and find that on average the byte error rate is 25.4% when sequencing 7 uniquely encoded and synthesized long DNA molecules of 2297 bp. This work provides the first study that has been done to directly compare soft and hard decoding performance of convolutional codes for the same population of nanopore reads. We show that Chandak *et al.*’s CTC decoding algorithm can significantly reduce byte error rate to 2.25% on average. However, because of the low throughput, we limited our analysis to just two encoded strands. We evaluated our new algorithm on this same sample of encodings and show that we provide comparable error rates (3.52%), but with a speedup of 257× compared to Chandak *et al.*’s soft decoder when evaluating both on GPU implementations. This speedup enables scaling to our full set of 7 strands to show that the error rate on this large sample is 2.59%. We then synthesized 10 additional strands spanning several lengths and encoding densities to understand the accuracy and density trade-offs. Based on our data, we project that our decoding can achieve read densities of 0.33 bits/base, 4× larger compared to coverage-optimized MSA approaches by Chen *et al.* (2021) (Supplementary Fig. S1). We also demonstrate our decoder’s flexibility by applying our algorithm to an open-source RNA basecaller, and we show that it achieves a lower byte error rate than DNA using the baseline HEDGES decoder with a state-of-the-art basecaller. This supports the feasibility of using RNA decoding as part of a data storage system.

## 2 Materials and methods

### 2.1 Information encoding

**Figure 1.**
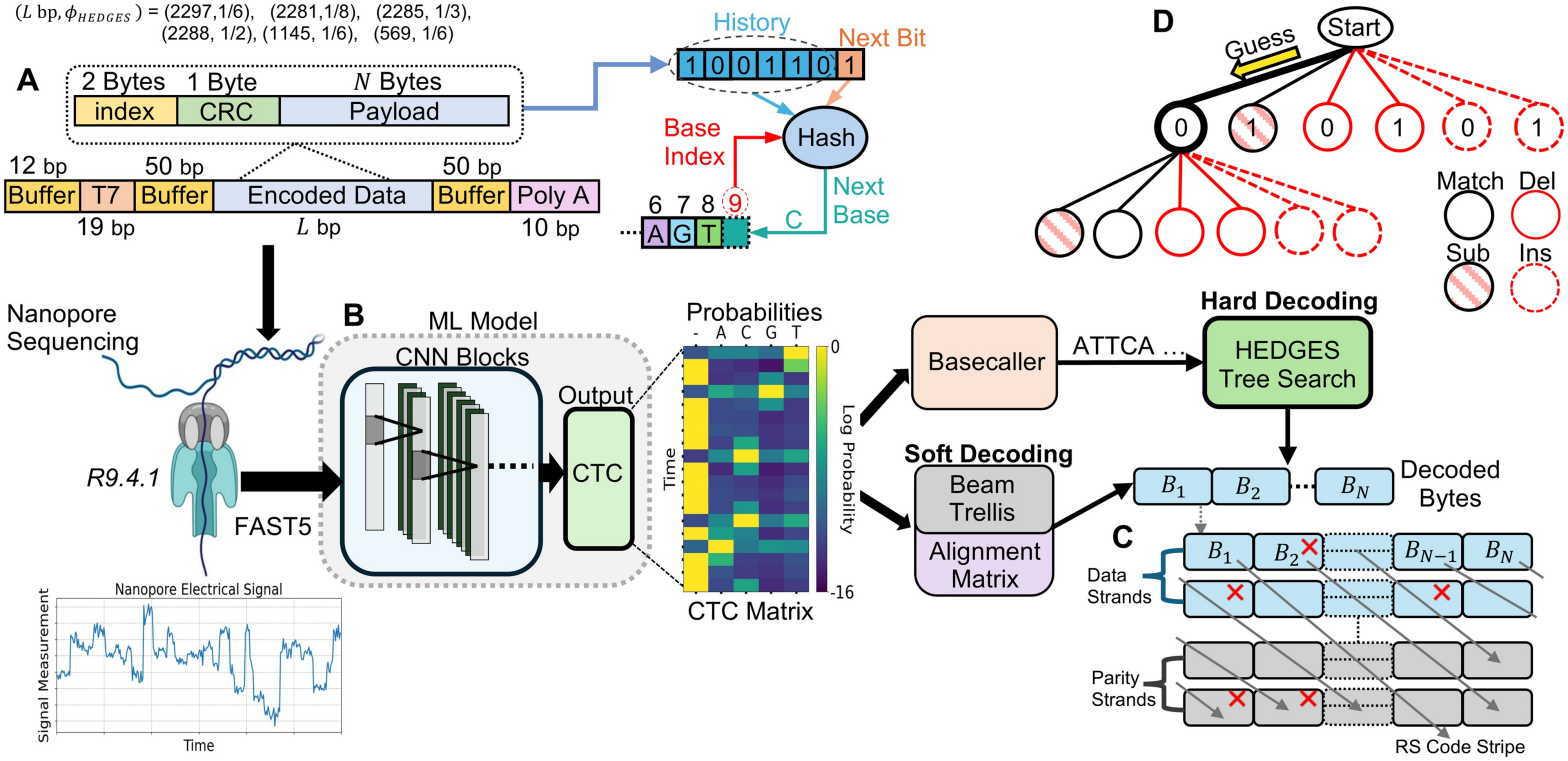
Overview of experimental workflow. (A) Encoding parameters and strand design used throughout the course of our experiments. (B) This work assumes a ML model that transforms nanopore signals to CTC outputs from which the code can be directly decoded (soft decoding), or decoded following a basecalling process that decodes the ML model output. (C) Diagonally striped RS outer code model assumed to allow for final densities to be calculated when taking into account the rate of byte-errors emitted from the studied decoders. (D) Outline of HEDGES decoding. Guesses are made on which bit was encoded which also emits a corresponding base according to the hash. Guesses include all possible error scenarios and are organized within a tree data structure.

We employ the HEDGES code as our baseline in this work (Press *et al.* 2020). We chose this encoding due to its ability to avoid repetitive bases, GC balancing constraints, and variable encoding densities. The HEDGES encoder builds a DNA strand based on the results of a hash algorithm that digests three pieces of information: *history bits*, *base index*, and the *next bit* to be encoded (Fig. 1A). The history bits are used in conjunction with the base index to embed the context of each encoded bit within the base sequence. The approach of combining history information during encoding places HEDGES within the class of convolutional codes. Such codes are decoded by making a series of guesses about what information was stored. Thus, the hash and embedded context is designed to generate distinct DNA sequences that can be distinguished even in the instances of errors injected by the channel as guesses are generated.

### 2.2 Soft decoding algorithms

Soft decoders leverage probabilities associated with each symbol to decide the most likely message sent given the received symbol probabilities. Such decoders are widely used and well known to offer advantages over hard decoding. While most prior work in DNA storage relies on hard decoding of sequencer generated basecall data, nanopore sequencing workflows make it possible to extract detailed per base probabilities. Nanopore basecalling workflows typically consist of two main steps: using a machine learning (ML) model to generate scores for assignment of bases to the electrical signal, and interpreting the scores to produce a final sequence of bases (Fig. 1B).

One ML model output commonly used for nanopore sequencing is the connectionist temporal classification (CTC) output (Neumann *et al.* 2022, Pagès-Gallego and de Ridder 2023). This output is formed as a matrix with two dimensions. One dimension being interpreted as time, and the other dimension corresponding to the *alphabet* that a message is constructed from. In this work, the alphabet is the four bases {A, G, C, T}. Each element of the matrix represents a log probability that a symbol of the alphabet or a *blank* occurs at a given CTC time step. The *blank* symbol is a special symbol in addition to the alphabet symbols that helps with determining probabilities of messages that have successive repeats of alphabet symbols. Based on the same CTC data, we consider two soft decoders that take different approaches to estimating message probabilities.

A key insight for CTC model outputs is to be able to learn and tolerate time variation in symbol signals (Graves *et al.* 2006). This has a natural application in nanopore sequencing considering dwell time variations that may occur as bases traverse the pore. Because of the time variation and probabilistic outputs, CTC outputs do not directly convey a single message. Instead, *CTC-encodings* are used to construct alignments of messages to the CTC data to calculate probabilities for the message. Such encodings allow for the representation of the same base symbol occupying multiple time steps, e.g. the encoded AAA decodes to a single base message A. However, to enable repeats in the decoded message, at least one blank (-) must be included to separate their CTC repeats from their decoded repeats. For example, the message AA is only allowed encodings of the form A-A.

The intuition behind both soft decoders in this work is to determine the message that best synchronizes with the CTC information by taking into account their different possible *CTC-encodings*. The soft decoder of Chandak *et al.* synchronizes messages by expanding the trellis complexity to evaluate message positions at every CTC time step. On the other hand, our approach considers and compares how well message prefixes align across all time steps, enabling a time and memory saving dynamic programming approach. As we will show, the approach taken to perform this synchronization significantly impacts the compute and memory complexity.

Leveraging CTC information is just one manner of using probabilistic information for decoding sequencing reads. Prior works such as Hamoum and Dupraz (2023) and Lenz *et al.* (2021) investigated utilizing error probabilities to improve the accuracy of convolutional decoders. However, Lenz *et al.* (2021) only consider a channel where insertions, deletions, and substitutions occur at a fixed rate. This is known to be an inaccurate representation for errors observed from nanopore sequencing due to their dependence on molecule composition as well showing the tendency to occur in bursts (Hamoum *et al.* 2021). While Hamoum and Dupraz (2023) recognizes this nuance and applies it to convolutional decoders for nanopore reads, their approach to gathering the probability information requires developing a statistical model by counting observed error patterns for each k-mer pattern. This approach could be sensitive to any changes to synthesis, sequencing technology, as well as changes to inference models. On the other hand, the approaches in this work derive probability information directly from the inference model’s output. This enables such decoders to be directly applicable to any newly trained model weights or architectures so long as the CTC output is maintained. Such flexibility is important as model configurations for nanopore sequencing is a quickly moving and expanding area of study (Pagès-Gallego and de Ridder 2023).

#### 2.2.1 Beam trellis algorithm

HEDGES was originally described with hard decoding, so we extend it to support soft decoding. Since it is a convolutional code, we construct a full *trellis* to represent its decoding steps. A trellis for HEDGES must have a width of at least $2^{H}$ states for *H* history bits. Traditionally, each state has two outgoing edges representing the transition to another history as new bits are added to a message. The next step is determining how to score each message with the CTC data while accounting its various *CTC-encoding* alignments.

**Figure 2.**
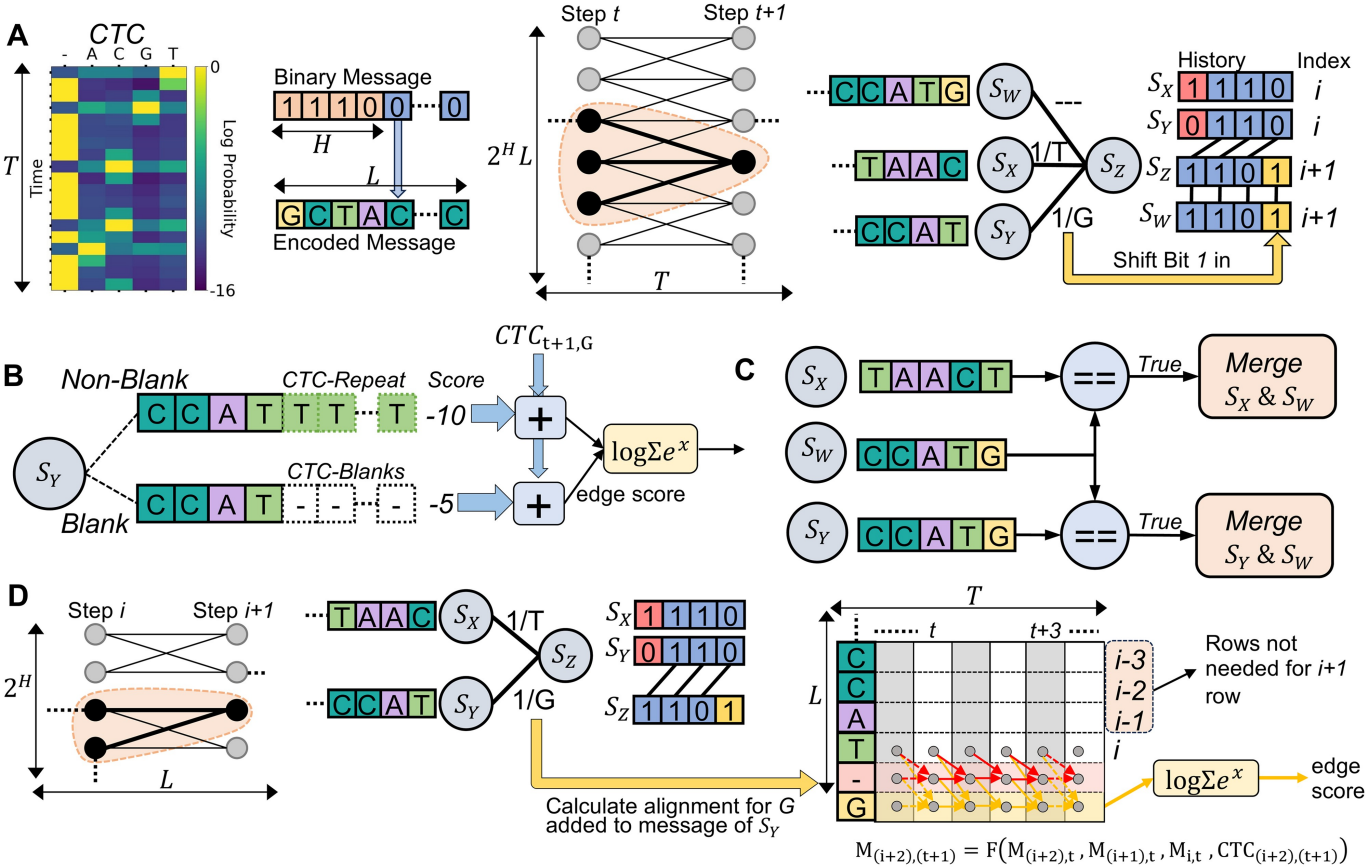
Soft decoding algorithms evaluated in this work. (A) The trellis architecture and connections of states used by the Beam Trellis algorithm (Chandak *et al.*). (B) Edge scoring mechanism of the Beam Trellis algorithm to account for *CTC-encodings* of candidate messages. (C) State merging required by the Beam Trellis algorithm to account for duplicate state messages. (D) Architecture and scoring methodology used by our novel alignment matrix algorithm. Arrows in the alignment matrix represent data used to calculate newly added rows for both the new blank and base symbols.

The approach of Chandak *et al.* (2020) to incorporating *CTC-encodings* into a trellis is to extend the number of states by a factor of the length of the encoded strand (*L*) for a total of $2^{H}L$ states (Fig. 2A). Now, each state represents a value of history at a given message index. In this approach, each state is updated a number of times equal to the time dimension of the CTC matrix (*T*). During the updating process for some state at trellis-step *t *+* *1 three candidates are considered from the previous step *t*. Two candidates advance the index of the decoded strand (*S_X_*, *S_Y_*), while the remaining *S_W_* does not (Fig. 2A). The state *S_W_* is the mechanism by which *CTC-encodings* that allow for a symbol to occupy multiple time steps are accounted for in decoding a fixed length message. Thus, every state carries a *non-blank* and *blank* score portion, which are combined together when calculating the total score for a transition (Fig. 2B). With multiple states advancing the decoded strand index differently, edges representing *CTC-encodings* that convey the same message may occur which requires that they are merged so that an accurate score for a message can be obtained (Fig. 2C). Given this algorithm’s similarities to so-called Beam search algorithms (Scheidl *et al.* 2018), we refer to this approach as the Beam Trellis.

The time complexity of this algorithm is $O(L^{2}T2^{H})$ because of the $L2^{H}$ number of states that are evaluated *T* times. The additional factor of *L* arises from the need to compare incoming messages of length *L* when evaluating each incoming edge for a state to determine if there are multiple *CTC-encodings* representing the same message. Likewise, since each state must store a complete message of length *L*, space complexity can be written as $O(2^{H}L^{2})$. Provided that *T* will grow proportionally to *L*, *T* factors can be replaced by *L* in the complexity expressions. From this, we can see that this algorithm has poor time and memory scaling as the length of messages increase.

#### 2.2.2 Alignment matrix algorithm

Our novel approach to integrating CTC information into a trellis is to calculate the alignment for each message of the $2^{H}$ states directly (Fig. 2D). This is done by using the algorithm of Graves *et al.* (2006) to calculate the so called forward variables that represent the total probability of a prefix for a message for a certain time step. Conceptually, each forward variable for a base in a message is stored in a matrix *M* of dimension *L *×* T*. Each element of *M*(*i*, *t*) representing the sum of probabilities of all *CTC-encodings* of L[0:i], the prefix of *L* up to and including position *i* for some time *t*. Thus, when transitioning from decoder step *i* to *i *+* *1 we can calculate M(i+1,t) for all *t*, e.g. row *i *+* *1 of *M*. Because this row represents the probability of each newly constructed message prefix at each time step, it can be used as a method to score each transition edge in the trellis. To transform the row of values into a scalar for comparison, we compute the log sum of exponentials over the log probabilities of the freshly computed row. Our reasoning for using this value is that it represents a total probability for a prefix across all times *t*.

In practice, to account for paths to a base that pass through blank symbols, a row for a blank is included in *M* previous to the newly added base as shown in Fig. 2D. Each of these newly added rows (blank and non-blank) can be completely derived from the most recent row corresponding to a base in the message. Thus, we do not store the entire matrix for every state or recalculate it every moment that it is needed. Instead we only store the most recently calculated row, and only perform computations needed to calculate the new row for a newly added base. In concrete terms, the row related to a base with blanks included is calculated as $M_{\left(i+2,t+1\right)}=\text{log}(e^{CTC_{t+1,i+2}}(e^{M_{i+2,t}}+e^{M_{i+1,t}}+e^{M_{i,t}})$. What this calculation represents is a summation of all probabilities for paths into the new base while accounting for the probability of the new base at the given time step as determined by the CTC matrix. This calculation based on Fig. 2D assumes that the base added by a trellis edge is different than the most recent base (G≠T). If this were not the case, the term $e^{M_{i,t}}$ is removed from the calculation because at least one blank character is needed as previously discussed to encode message repeats.

The time complexity of our algorithm is $O(2^{H}LT)$. We determine this given that the trellis has $2^{H}$ states and *L* trellis evaluation steps. At each evaluation, we must calculate an alignment across *O*(*T*) time steps for each edge incoming to each state. Assuming the number of edges and the number of bases added to a message on each transition is fixed for a given code we assume these factors to be *O*(1). Thus, the time complexity of a single trellis propagation step is $2^{H}T$, and the complexity of $O(2^{H}LT)$ follows. This complexity is made possible by the dynamic programming approach taken to calculating rows for new bases, rather than re-calculating rows already visited.

Memory complexity follows a similar argument, where we only store 1 row of a matrix for each state so that alignments can propagate. Thus, a memory complexity of $O(2^{H}T)$ is achieved for our algorithm. Assuming *T* is proportional to *L*, our algorithm reduces complexity by a factor of *L*. A detailed pseudo code description of the Alignment Matrix algorithm can be found in Supplementary Section G.

#### 2.2.3 GPU parallelization

We recognize that to perform all of the necessary calculations across each trellis approach, significant computational effort will be required to aligning long messages with their correspondingly long CTC matrix. Provided the abundance of independent calculations that can be performed across states of the Beam Trellis algorithm and the rows of the matrix in the Alignment Matrix algorithm, we leverage GPUs to accelerate each algorithm. In our implementations, we strive to utilize best practices by considering occupancy, shared memory resources, and memory access coalescing patterns. Details of our GPU implementations for both soft decoders can be found in Supplementary Section A.9.

When benchmarking soft decoder algorithms, they are run on nodes consisting of a NVIDIA RTX 2060 Super GPU device, a single AMD EPYC 7302P 16-Core processor, and 128GB DDR4 DRAM. The baseline HEDGES decoder runs on either Intel Xeon Gold 6226 or 6130 processors that have 192 GB RAM per node.

### 2.3 Experiment workflow

#### 2.3.1 Encoding information in molecules

To complete our evaluation of decoders, we encode and synthesize 17 unique DNA template molecules following Fig. 1A. A primary goal for our analysis is to understand how information error rates will be influenced by the rate of the encoding and the length of a molecule passing through a pore. Thus, our designs cover HEDGES code rates of $\frac{1}{8},\;\frac{1}{6},\;\frac{1}{3}$, and $\frac{1}{2}$. Each encoded strand was bookended by signal buffer sequences of length 50 bp to protect information carrying bases from transient behaviour entering and leaving the nanopore. On the 3′ end we also include an additional 10 base poly A tail, and on the 5′ end we allocate 19 bp for a T7 promoter to allow for transcription of RNA molecules and an additional 8 bp for a synthesis buffer. For every design, we keep these additional 5′ and 3′ bases constant. The full length of the $\frac{1}{6}$ rate strands with additional regions is 2297 bp. For the rates of $\frac{1}{8},\;\frac{1}{3}$, and $\frac{1}{2}$, we aim to keep strand length relatively constant with their respective entire lengths being 2281, 2285, and 2288 bp. For the $\frac{1}{6}$ rate, we synthesize 2 additional length of molecules that are 1145 and 569 bp to study short length strand impacts on nanopore sequencing. For HEDGES parameterization, we limit homopolymers to be a maximum of 3 and fix a GC content of 50% over 12 base pair windows. Each strand was ordered as DNA gBlocks Gene Fragments from Integrated DNA Technologies.

Our molecules are derived from several sources of data. The strands for the $\frac{1}{6}$ HEDGES rate and 2297 bp design are all derived from the same thumbnail image of the periodic table symbol for phosphorous. The $\frac{1}{8},\;\frac{1}{3}$, and $\frac{1}{2}$ HEDGES rates strands encode the complete 8th, 4th, and 6th amendments of the Constitution of the United States, respectively. The 1145 and 569 bp with $\frac{1}{8}$ HEDGES rate designs encode the first 76 characters and characters 28–56 of the 4th amendment respectively. Copies of the raw encoded data and exact strands synthesized for these experiments are included within our public code release.

#### 2.3.2 Nanopore sequencing and preprocessing

With our synthesized DNA molecules, we sequence all strands using ONT nanopores of version R9.4.1. For each sequencing run, we use the latest available ONT basecalling models at the time of sequencing as reported in Supplementary Table S1 to generate FASTQ information. We used this initial FASTQ data in order to demultiplex individual reads to their original encoded strand and to eliminate reads that we do not want to impact measured decode rates from our decoders. Demultiplexing is done via the encoded index and CRC bytes within the encoded strand (Fig. 1A). Further details are available in Extended Methods A.

Using HEDGES to decode basecalls, we attribute each read to an encoded strand if the decoded index bytes is in agreement with the CRC byte. While performing read attribution, we eliminate short reads and exceedingly long chimeric reads (Supplementary Fig. S2). Our reason to exclude these reads is to obtain a clearer understanding of decoder performance on the characteristic of nanopore signals and not information loss that may occur from small fragments that result from other molecular handling and processing steps. We verify that our preprocessing does not bias the quality of reads to significantly higher qualities, and eliminated reads correlate to outlier low quality reads that would be considered failed reads by ONT (Q Score<9). When considering reads of Q Score>9Supplementary Fig. S5 shows when demultiplexing a 5 strand sequencing run of $\frac{1}{6}$ rate 2297 bp strands the average Q Score of retained reads varies between 13.37−13.57 compared to 13.25 of the entire sequencing run.

To analyze decoding accuracy performance and basecall error rates while limiting computational overhead, we randomly sample our set of attributed reads to sizes appropriate for our analysis needs. To ensure high confidence in basecall error rates and error patterns, we take a subset of 100k reads for the baseline hard decoder. We analyze decoder performance and basecall error patterns for the chosen 100k subset against the original ONT basecall FASTQ. We also collect the FAST5 data corresponding to this subset so that basecall information of the CTC model used for soft decoding can be analyzed (Fig. 1B). We use the latest CTC version of the open source ONT Bonito basecaller available, and the exact code version of this model is included in our code release repository. For soft decoding, we are only interested in byte error rate and so we reduce the number of samples. For our novel soft decoder, we take a 10k subset of reads from the original 100k subset for each strand. In our evaluation of Chandak *et al.* (2020)’s CTC decoder we experienced slow throughputs caused by the complexity of the decoder. Thus, we use a 2k subset sampled randomly from the 10k set.

#### 2.3.3 CTC data orientation and buffer regions

CTC matrices received from the ML model will not only include data related to the payload but also buffer regions. Leaving data within the CTC matrix related to buffer regions can potentially disrupt the soft decoding algorithms by causing alignments of payload bases to signals that are unrelated. Furthermore, DNA molecules can be sequenced as their encoded forward versions or their reverse complement. This is important to consider so that the decoder can generate the appropriate bases in the trellis. To solve this problem with only CTC information, we adapt the techniques of Kürzinger *et al.* (2020) to locate and trim buffer region CTC data (Supplementary Section A.8).

#### 2.3.4 Measuring byte error rate and system density

For all bytes within each strand, we calculate the byte error rate as the total number of decoding failures observed for each individual byte. To account for biasing that occurs in HEDGES decoding where bytes toward the end of a strand have a higher failure rate (Supplementary Fig. S12), we calculate a mean rate (${\bar{P}}_{B}$) for byte errors across all positions and encoded strands (Supplementary Equation S3).

Because the rate ${\bar{P}}_{B}$ will be > 0, supplemental error correction is needed to resolve byte errors following HEDGES decoding. Reed Solomon (RS) codes have be shown to be able to overcome errors within the DNA storage channel, especially errors related to strand less (erasures) by generating RS codewords with information *across* encoded sequences (Grass *et al.* 2015, Organick *et al.* 2018, Press *et al.* 2020). With inter-strand redundancy, we can resolve residual errors that remain after HEDGES, an approach that Press *et al.* (2020) take as well.

Following Press *et al.* (2020), we use ${\bar{P}}_{B}$ to model the effective error rate that is observed in an RS codeword with diagonally striped bytes between strands (Fig. 1C). Using ${\bar{P}}_{B}$, we calculate the probability to decode an RS codeword (Supplementary Equation S10) and choose a design that can access 1 TB with a mean time to failure (MTTF) of 10^6^ accesses. With the outer code design, we calculate a complete density, ϕ, in bits per base following the steps of Supplementary Section A.5.

## 3 Results

### 3.1 Hard decoding byte errors

**Figure 3.**
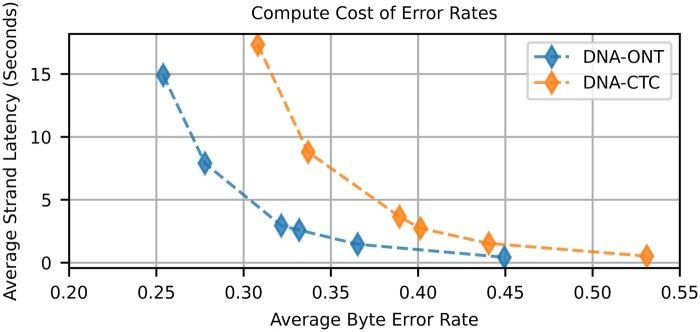
Average strand decode time versus average byte error rate when using the HEDGES baseline hard decoding algorithm for ONT and CTC basecalling models.

When considering the baseline hard decoder of the HEDGES code, we want to understand both the rate bytes can be recovered and also the computational effort expended for a given byte error rate. As shown in Fig. 1D, the HEDGES decoding algorithm forms a tree representing guesses that can be made on what information was encoded and what errors may have occurred within the basecalled sequence. Figure 3 shows the relationship of compute time and byte error rate for the two basecallers considered where *DNA-CTC* is the CTC output-based model used as the basis of our soft decoder algorithms, and *DNA-ONT* represents ONT production basecallers (Supplementary Table S1). Both basecallers are applied to the same subset of 100k reads for all seven strands of the 2297 bp $\phi_{\mathrm{HEDGES}}=\frac{1}{6}$ design.

We find that for both basecallers, there are diminishing returns when allocating more guesses to the algorithm, indicating that it is intractable to reach low byte error rates by simply increasing compute effort. The lowest mean byte error rates achieved for the ONT and CTC models were measured to be 0.254 and 0.308, respectively, when allowing a limit of 6 million guesses. When placing these byte error rates in our approach to determining overall system density, we find that hard decoding error rates cannot meet our target $\text{MTTF}=10^{6}$ for systems larger than 1 TB (Supplementary Fig. S13). The higher byte error rate of decoding the CTC basecaller also serves as a control to show that the CTC model does not provide an unfair advantage in the quality of information produced by the ML model compared to the ONT models. Corresponding to this higher byte error rate, we find that CTC basecaller base error rates are 7.6% on average and are higher than the ONT basecallers base error rates (5.63%, Supplementary Figs S3 and S4).

### 3.2 Soft decoding performance analysis

**Figure 4.**
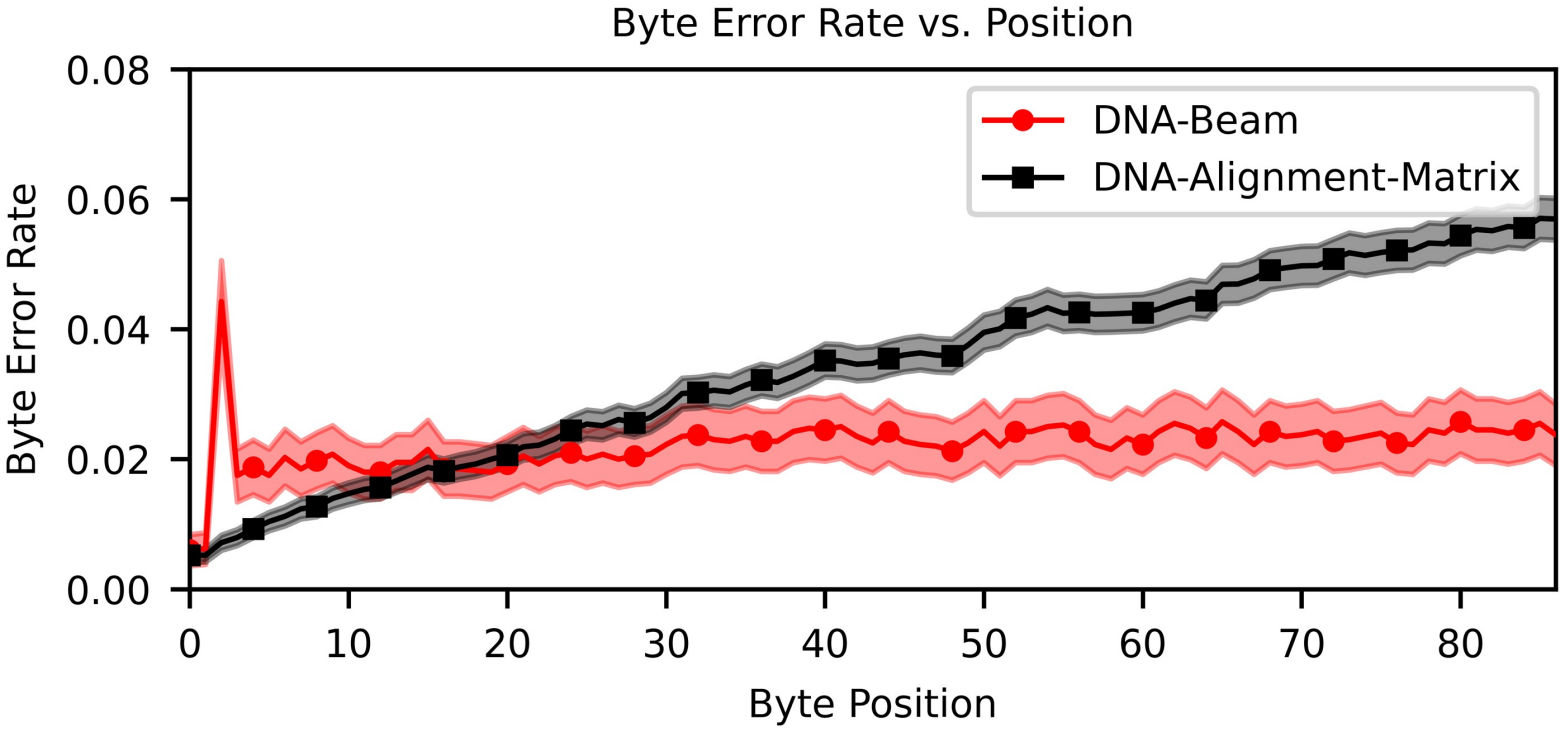
Byte error rate versus byte position for both soft decoders. Points represent mean error rate across encoded strands (Supplementary Equation S2), error bars calculated with Supplementary Equation S5 estimate a 95% confidence interval.

Table 1 reports key metrics when comparing all three decoding algorithms. Error rates and densities in this table are derived from a subset of two of the seven 2297 bp $\phi_{\mathrm{HEDGES}}=\frac{1}{6}$ design strands. Our analysis demonstrates that for the same encoded strands, the Beam Trellis algorithm can reduce ${\bar{P}}_{B}$ by an order of magnitude compared to hard decoding using 6 million guesses. We also find that our novel Alignment Matrix algorithm greatly improves ${\bar{P}}_{B}$ on this data set to 3.52%. The slight increase in byte error rate is a product of the error profile of byte errors within a strand as shown in Fig. 4. We find that the byte error rate of the Beam Trellis algorithm remains relatively constant across the length of the strand, while the bytes at the end of a strand have higher error rates when decoded by the Alignment Matrix algorithm. This is caused by error cascades that are generated by the prefix probability scoring metric from data dependencies in the matrix we use to store alignments in Fig. 2D. If a base is chosen such that it negatively impacts the score of successive bases that represent the correct path through a trellis, then it can become difficult to build a high enough score to correct wrong path choices when only a few bases at most are added to messages between comparisons (Supplementary Fig. S9). On the other hand, the Beam Trellis algorithm has the flexibility to adjust states representing message indexes to best align to the CTC matrix.

**Table 1.** Comparison of decoding metrics for algorithm and molecule combinations.^a^

Molecule	Algorithm	*T*_read_ s/read	${\bar{P}}_{B}$ (%)	ϕ bits/bp	Error-free reads (%)
DNA	Alignment matrix	0.713	3.52	0.203	87.15
	Beam	183.17	2.25	0.221	84.08
	Tree-6M (hard)	12.78	23.44	0.0128	58
	Tree-1M (hard)	2.7	29.74	N/A	49.5
RNA	Alignment matrix	1.06	12.52	0.106	74.34
	Tree-6M (hard)	16.29	23.61	0.0128	56.72
	Tree-1M (hard)	3.46	32.62	N/A	43.72

a $T_{\text{read}}$ measures just decode time excluding CTC model time. All hard decoders use ONT basecaller models.

**Figure 5.**
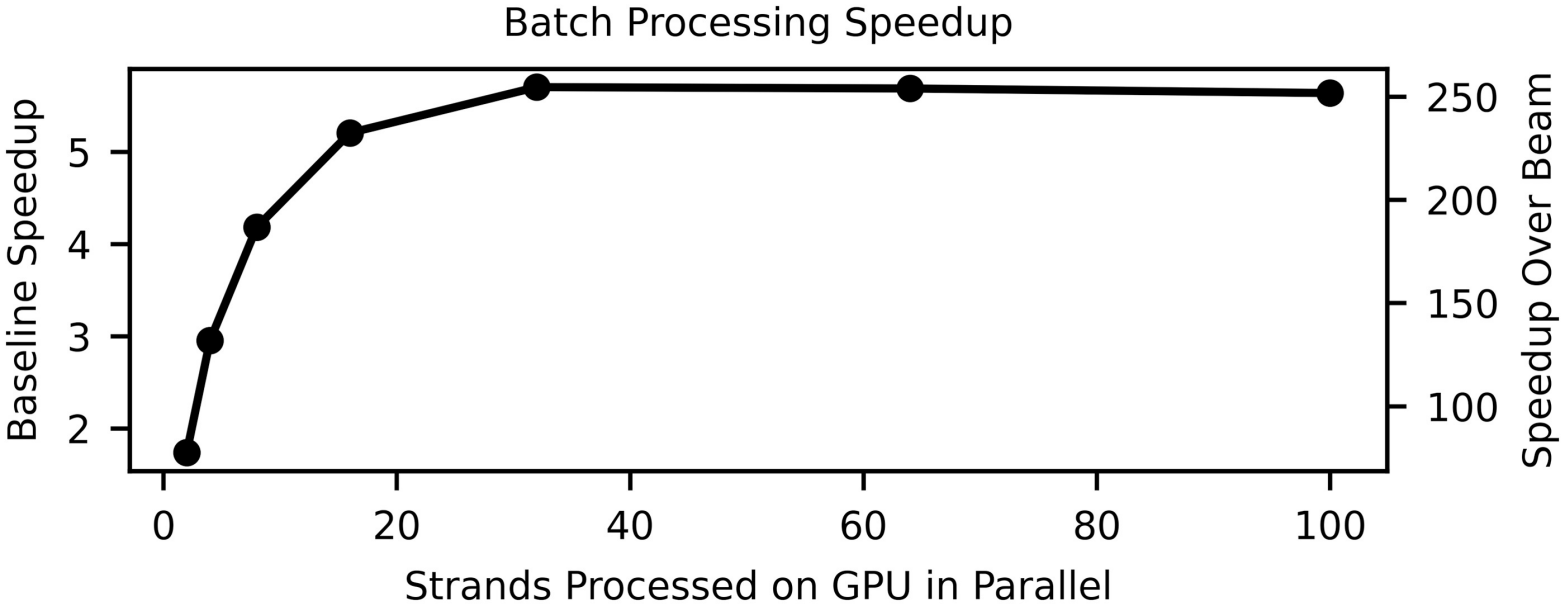
Speedups achieved with alignment matrix algorithm when batching multiple reads for GPU computation compared to decoding a single read (left *y*-axis) and compared to the decode rate of Beam Trellis algorithm (right *y*-axis).

While the Beam Trellis algorithm does decrease ${\bar{P}}_{B}$ by 39% compared to the Alignment Matrix algorithm, in practice the impact on density is quite small (8.8% increase). Furthermore, the Alignment Matrix algorithm has a 257x larger throughput when we benchmark on a direct comparison of decoding 400 CTC matrices that we extracted from their respective reads. Our large gains in performance are derived from two factors. First, the computational complexity of our algorithm is reduced by a factor of *L*, and given we are decoding long messages, this can significantly impact the practical performance of the algorithms. Second, the limited complexity of our trellis architecture enables batching multiple reads to increase GPU utilization (Fig. 5).

For this strand design, the Alignment Matrix algorithm launches 3072 threads per read (Supplementary Fig. S9). Given the total thread occupancy of our GPUs ($32\frac{\text{warps}}{\text{SM}}\times 32\frac{\text{threads}}{\text{warp}}\times 34\frac{\text{SM}}{\text{GPU}}=34.8$k threads), latency can be hid between data dependencies in the matrix by increasing the number of parallel reads decoding. However, for the Beam Trellis algorithm, we do not consider batching given that we launch approximately 552k threads per read which already saturates GPU thread occupancy (Supplementary Fig. S8).

We also perform an analytic estimation of the total memory footprint of the two soft decoders (Supplementary Fig. S10). We found that for the 400 reads used for benchmarking, the amount of memory used for each read on average is 52x lower when using the Alignment Matrix algorithm (0.032 GB) versus the Beam Trellis algorithm (1.66 GB).

Using the same two strands, we demonstrate the versatility of soft decoding CTC data by applying our decoder library with no changes directly to the CTC outputs of the open source RODAN RNA basecaller (Neumann *et al.* 2022). Table 1 shows that again, CTC soft decoding outperforms hard decoding even when using ONT production basecallers. While we do find RNA has about 15% less total error free reads compared to DNA soft decoding, these results imply that storage systems that rely on RNA are feasible even under the conservative single-read assumption.

### 3.3 Optimizing alignment matrix parameters

Given the computational advancements made by Alignment Matrix algorithm, it is now possible to evaluate and understand parameter choices for the decoder. The parameters that we consider are strand length, HEDGES encoding density, and the reads chosen to provide to the decoder. We choose strand length to understand how the computational complexity of the decoder impacts decode time in practice. Also, given the positional dependence on error rate for bytes, we are interested in understanding how this profile may change with shorter strands. When changing the encoding rate, we want to understand the density tradeoff of higher density encodings with the corresponding byte error rate that must be designed around. Lastly, we consider if information density can be significantly influenced by the quality of reads given to the decoder.

Table 2 summarizes decode throughput, ${\bar{P}}_{B}$, and ϕ for each design and when we consider reads of all quality in our data set (Nominal) and when reads are chosen from the set of reads with Q Scores in the range [15.1,15.4]. Decoding rate as a function of strand length shows that the rate of information decoded outpaces the rate of information lost from a strand when shortening strands. For example, the $569\;\text{bp},\frac{1}{6}$ design has 383 bases of information after removing buffers and indexing and decodes at 0.0719 s read, while the $2297\text{bp},\frac{1}{6}$ design encodes information over 2112bp and decodes at 0.723 s/read. So, while the shorter strand needs 5.5x as many strands to encode the same information, we can infer that decoding this total amount of information is 1.8x faster. Comparing ${\bar{P}}_{B}$ for shorter strands shows that when there are less bytes within a strand the overall byte error decreases by reducing impacts of cascaded errors (Supplementary Fig. S15). However, the overall density (ϕ) is largest for the $2297\;\text{bp},\frac{1}{6}$ design because it can amortize overhead related to indexing and buffer regions more efficiently. These results indicate that strand length may be tuned to maximize density or byte throughput.

**Table 2.** Table of timing benchmarking, byte error rate (${\bar{P}}_{B}$), and density for six strand designs that are encoded and synthesized for experimental evaluation.^a^

Design		Nominal		Q score [15.1,15.4]	
$L(\text{bp}),\phi_{\text{HEDGES}}$	*T*_read_ s/read	${\bar{P}}_{B}$ (%)	ϕ bits/bp	${\bar{P}}_{B}$ (%)	ϕ bits/bp
2281,1/8	0.645	1.27	0.175	0.92	0.181
2297,1/6	0.723	2.59	0.215	1.12	0.241
1145,1/6	0.209	1.77	0.203	0.93	0.218
569,1/6	0.0719	0.81	0.163	0.29	0.174
2285,1/3	0.761	22.55	0.041	10.17	0.262
2288,1/2	0.82	56.34	n/a	37.18	n/a

a *Nominal* refers to the case where each strand in each design is evaluated over a 10k read set derived from the entire space of viable sequencing reads. Q score refers to analysis done when considering a subset of 10k reads that all have Q scores in the range of 15.1 and 15.4. Seconds/read includes both ML model and decoder time, and decoding is done with a batch size of 50.

When comparing changes in $\phi_{\text{HEDGES}}$, we find that between any two encoding densities the lower density has a lower ${\bar{P}}_{B}$. However, this does not always lead to higher densities. For example, $\phi_{\text{HEDGES}}=\frac{1}{8}$ has ${\bar{P}}_{B}=1.27\%$ and $\phi_{\text{HEDGES}}=\frac{1}{6}$ has ${\bar{P}}_{B}=2.59\%$, but the density for the former is 0.175 bits/bp while $\phi_{\text{HEDGES}}=\frac{1}{6}$ can achieve a 0.215 bits/bp with its error rate. However, ${\bar{P}}_{B}$ becomes too large to build efficient RS codes for the remaining higher encoding densities of $\phi_{\text{HEDGES}}=\frac{1}{3},\frac{1}{2}$. We consider if passing better quality reads can increase the viability of larger $\phi_{\text{HEDGES}}$ by evaluating 10k reads in 15 Q-score bins ranging from 10.9 to 15.1. By controlling the Q score, we show that ${\bar{P}}_{B}$ can be reduced greatly for $\phi_{\text{HEDGES}}=\frac{1}{3}$ from 27% for reads in the Q score range [10.9−11.2] to 10.17% for reads in the range of [15.1−15.4] (Supplementary Fig. S16). This leads to a 6.4x increase in density for $\phi_{\text{HEDGES}}=\frac{1}{3}$ when higher quality reads are given to the decoder (Table 2).

**Figure 6.**
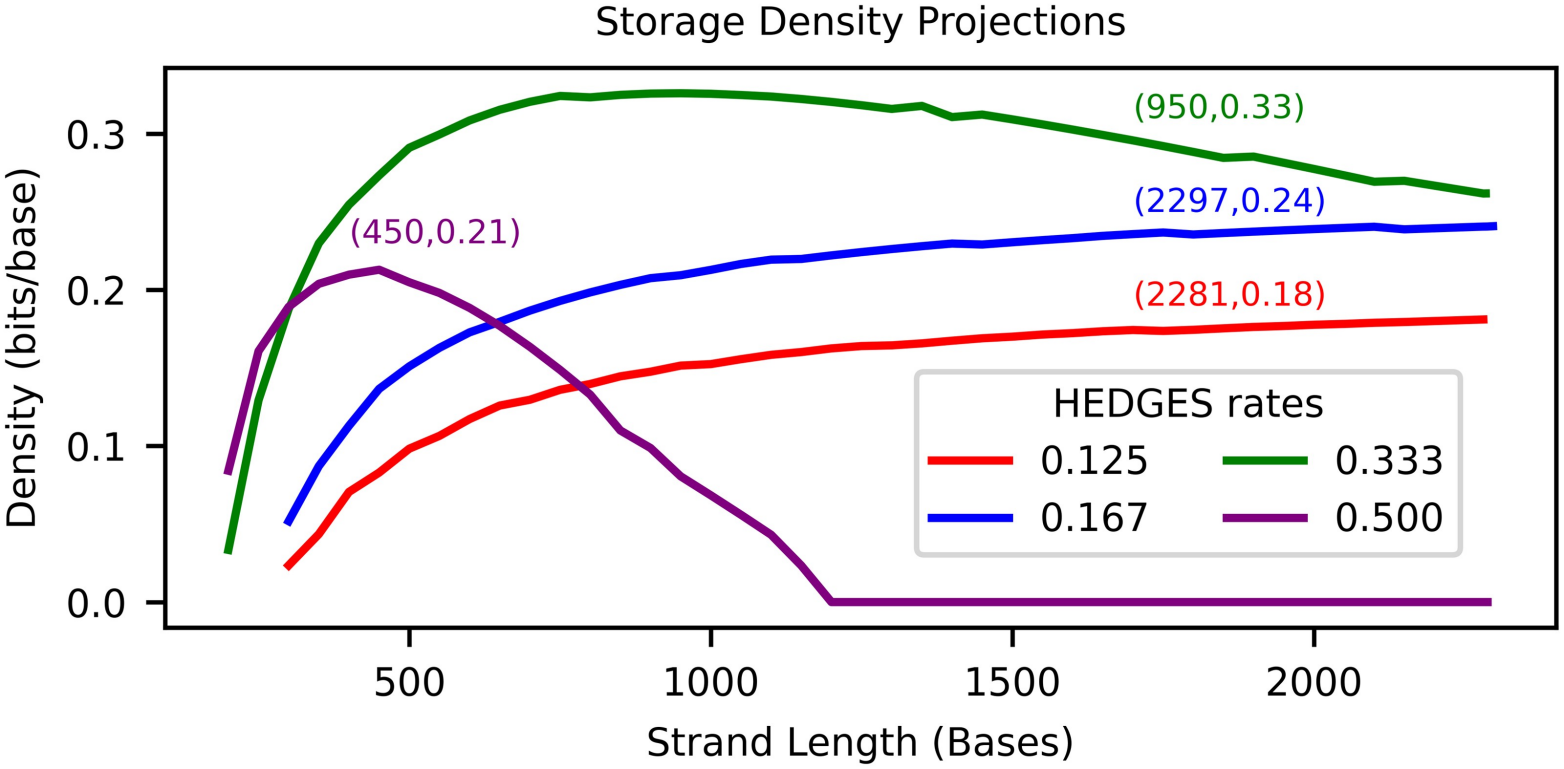
Projected densities when optimizing parameters of the alignment matrix algorithm.

We combine our findings together in Fig. 6 by tuning strand length for each code rate when assuming error rates are for Q scores in the range of [15.1−15.4]. This allows for projections to be made about the maximum density that can be achieved with our decoder. In this analysis, we make the assumption that the measured error rate versus byte position can be truncated to emulate shorter strand lengths than what were synthesized. This analysis shows that the largest ϕ=0.33 bits/bp is reached for $\phi_{\text{HEDGES}}=\frac{1}{3}$ and strand length of 950 bp. Compared to the highest read density of Supplementary Fig. S1 (Chen *et al.* 2021), this projected density is 4x larger. These curves also indicate that limiting ${\bar{P}}_{B}$ with shorter strands for larger encoding densities is important to maximize density, but for $\phi_{\text{HEDGES}}=\frac{1}{6},\frac{1}{8}$ their ${\bar{P}}_{B}$ is low enough such that longer strands are preferred in order to amortize the overhead of bases associated with indexing or overhead for functional sites.

## 4 Conclusion

Most DNA storage systems remain at a scale <1 GB and can tolerate slow decoding. However, to scale to large capacities and to advance our understanding of these systems, decoders must improve their throughput, read density, and support for varying strand length, molecule type, and encoding density. Our decoder is versatile and a step in that direction, but not the final step.

Further improvements in speed and error rate are needed and highly possible. Porting to more powerful GPUs will deliver speedups proportional to their threading capacity since Alignment Matrix is largely compute-bound and has only modest memory needs. Soft decoding is a key reason for low error rates, and we demonstrated Alignment Matrix works on outputs from both Bonito and RODAN on DNA and RNA, respectively. This implies that our approach is able to work independently of a particular model. We expect that our approach can benefit from advances in basecaller models as they are released. However, to ensure they remain compatible in the long run, it may be important to adapt to other common model outputs such as conditional random fields (Pagès-Gallego and de Ridder 2023). Additionally, the learned model could be coupled with the codeword space by leveraging application-specific training to improve inference quality of strands specific to the encoding (Wick *et al.* 2019).

## Supplementary Material

btaf006_Supplementary_Data
