# Supplementary material for: Nanopore decoding with speed and versatility for data storage
Source: Bioinformatics. 2025 Jan 8;41(1):btaf006. doi: 10.1093/bioinformatics/btaf006 (PMC11755093; doi:10.1093/bioinformatics/btaf006)
Supplement: btaf006_Supplementary_Data [file btaf006_supplementary_data.zip › b957f_supplemental_manuscript.pdf]

A. Extended Methods

A.1. MSA Analysis

Supplemental Figure 1 provides an overview of read and write densities that are achieved by prior works that use MSA for decoding nanopore sequencing reads. We calculate read density by considering both the write density and the sequencing coverage used that each point is labeled with. Given that the write density is the base rate in which information is represented in terms of bases, and coverage represents on average the number copies of each encoded base, we then calculate read density as simply write density/coverage.

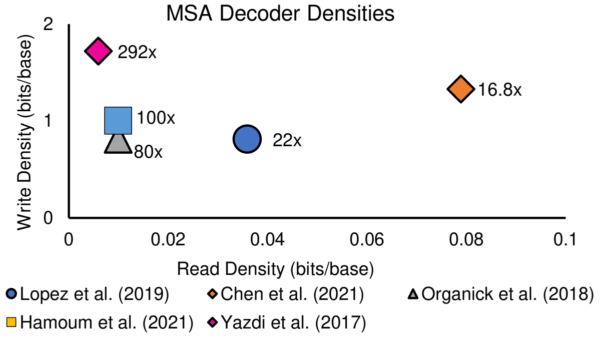

Fig. 1: Read/write information densities of MSA decoders used for nanopore sequencing.

A.2. Synthesis, Sequencing, and Transcription

A.2.1. Ordering and reconstitution

All designed strands were ordered as DNA gBlocks Gene Fragments from Integrated DNA Technologies (IDT). Upon arrival, dried fragments were reconstituted in either nuclease-free water or IDTE pH 8.0 (IDT, 11-05-01-13) and incubated at 50°C for 20 minutes. Reconstituted DNA fragments were stored at 4°C until use.

A.2.2. *In vitro* transcription (IVT)

IVT was performed for each strand separately with HiScribe T7 Quick High Yield RNA Synthesis Kit (NEB, E2050) according to manufacturer's recommendations. Briefly, DNA fragments (70-100 ng) were mixed with 10 μL of NTP Buffer Mix, 2 μL T7 RNA Polymerase Mix, and nuclease-free water. In some reactions, 1 uL of 100 mM DTT was added due to a change in the manufacturer's protocol. Reactions were then incubated at 37°C for 2 hours. Next, template DNA was removed by adding 30 μL of nuclease-free water and 2 μL DNase I (NEB, M0303S) to each reaction followed by incubation at 37°C for 15 minutes. RNA was then purified using the Monarch RNA Cleanup Kit (NEB, T2050L) following manufacturer's recommendations. RNA was eluted in 50 μL nuclease-free water and yield was quantified using Qubit RNA high sensitivity assay (Thermo Fisher Scientific, Q32852). RNA size was quantified using either RNA ScreenTape (Agilent, 5067-5576) or high sensitivity RNA ScreenTape (Agilent, 5067-5579) run on a TapeStation 4200 (Agilent, G2991BA). RNA was stored at -80°C until sequencing.

A.2.3. Nanopore direct RNA sequencing

RNA strands were either sequenced individually or pooled at equimolar amounts by length and encoding (Supplementary Table 1). Samples were prepared for direct RNA sequencing using the Oxford Nanopore Technologies (ONT) direct RNA sequencing kit (ONT, SQK-RNA002) and protocols provided by ONT (protocol version: direct-rna-sequencing-sqk-rna002-DRS_9080_v2_revO_14Aug2019-minion). Briefly, RT Adapter (ONT, SQK-RNA002) was ligated to 500 ng of each individual RNA sample or pool using T4 DNA ligase (NEB, M0202) for 15 minutes at room temperature. Reverse transcription was then performed with SuperScript III Reverse Transcriptase (Thermo Fisher Scientific, 18080044) by incubation at 50°C for 50 minutes, 70°C for 10 minutes, and then a 4°C hold. Reverse transcription reactions were cleaned up using 1.8x RNAClean XP beads (Beckman Coulter, A63987) and 70% ethanol. Reverse transcribed samples were eluted in nuclease-free water and quantified using Qubit 1x dsDNA high sensitivity assay (ThermoFisher Scientific, Q33230) or Qubit dsDNA high sensitivity assay (Thermo Fisher Scientific, Q32851). Next, reverse transcribed samples were ligated to the RNA Adapter (ONT, SQK-RNA002) using T4 DNA ligase (NEB, M0202) at room temperature for 15 minutes and then purified using 1x RNAClean XP beads and Wash Buffer (ONT, SQK-RNA002). Final libraries were eluted in 21 uL Elution Buffer (ONT, SQK-RNA002) and quantified using Qubit 1x dsDNA high sensitivity assay or Qubit dsDNA high sensitivity assay. Each library was sequencing on a R9.4.1 MinION flow cell (ONT, FLO-MIN106D) run on a GridION Mk1 sequencing device (ONT, GRD-MK1). Details about software, basecallers, and basecalling models used can be found in Supplementary Table 1.

A.2.4. Nanopore DNA sequencing

DNA strands were either sequenced individually or pooled at equimolar amounts by length and encoding (Supplementary Table 1). Samples were prepared for nanopore DNA sequencing using the LSK110 ligation sequencing kit (ONT, SQK-LSK110) and protocols provided by ONT (protocol version: genomic-dna-by-ligation-sqk-lsk110-GDE_9108_v110_revV_10Nov2020-minion).

Briefly, 200 fmol of individual DNA fragments or pools were mixed with 3 μL NEBNext Ultra II End Prep Enzyme Mix and 3.5 μL Reaction Buffer (NEB, E7180S) and incubated at 20°C for 5 minutes followed by 65°C for 5 minutes to repair fragment ends. DNA was then purified using 1x AMPure XP beads (Beckman Coulter, A63881) along with 70% ethanol, eluted in 61 μL nuclease-free water at room temperature, and quantified with the Qubit 1x dsDNA high sensitivity assay. End-prepped DNA was then mixed with 25 μL Ligation Buffer (ONT, SQK-LSK110), 10 μL NEBNext Quick T4 DNA ligase (NEB, E7180S), and 5 μL Adapter Mix F (ONT, SQK-LSK110). Reactions were incubated for 15 minutes at room temperature. DNA was purified using 1x AMPure XP beads and Short Fragment Buffer (ONT, SQK-LSK110). DNA was eluted in 15 μL Elution Buffer (ONT, SQK-LSK110) at 37°C for 10 minutes. DNA was then quantified with the Qubit 1x dsDNA high sensitivity assay. Each library (50 fmol) was sequencing on a R9.4.1 MinION flow cell (ONT, FLO-MIN106D) run on a GridION Mk1 sequencing device (ONT, GRD-MK1). Details about software, basecallers, and basecalling models used can be found in Supplementary Table 1.

A.3. Calculating Byte Error Rate

We calculate the byte error rate $P_{l,s}$ for a given byte position l in an encoded strand s that with $|R|$ sequencing read samples as:

$$P_{l,s} = \frac{\sum_{r=0}^{|R|_s} \mathbf{I}_{l,s,r}}{|R|_s} \tag{1}$$

Where $\mathbf{I}_{l,s,r}$ is an indicator function that evaluates to 1 when there is a different byte compared to the original data in read r at byte position l for strand s, and 0 otherwise. To aggregate the byte error rates across encoded strands to determine general trends for a given design across byte positions, we take the arithmetic mean of Equation 1 across all strands ($|S|$) in a design as follows:

$$P_l = \frac{1}{|S|} \sum_{s=0}^{|S|} P_{l,s} \tag{2}$$

When considering a total aggregate byte error rate over all positions and encoded strands to determine a singular error rate that can be used to calculate outer code requirements we average Equation 1 over both the total number of positions $|L|$ and the strands that have been encoded for a given design:

$$\overline{P}_B = \frac{1}{|L|} \sum_{l=0}^{|L|} \frac{1}{|S|} \sum_{s=0}^{|S|} P_{l,s} \tag{3}$$

Equation 3 can be interpreted as an estimate of the byte error rate that would be generated if all bytes of all encoded strands and positions were equally likely to be chosen at random assuming that each position and strand are equally represented in the population. Thus, it represents the amount of information that can be recovered on average for each byte and indicates the information capacity of the channel for a given combination of inner code and decoder. This in turn can be used to determine additional redundancy for the inner code that is necessary to ensure a high reliability for recovering a data set of a certain number of bytes.

A.4. Byte Error Rate Measurement Error

Given that we take a random sample of reads to generate a data set that can make computational times reasonable, our measurements of $P_{l,s}$ are thus a random variable. The value of $P_{l,s}$ is a sample of the mean of the Bernoulli random variable $\mathbf{I}_{l,s}$ that is 1 when an error occurs in strand s at position l. We assume that all $P_{l,s}$ are normally distributed given our methodology for sampling strands. A general rule that allows the normal distribution assumption to be made for the proportion is that $np \geq 5$ should be satisfied for n samples and a proportion p. Given the error rate is the order of 10% and number of samples for the baseline HEDGES decoding algorithm is 100k, the $np \geq 5$ threshold is easily met. `Beam Trellis` algorithm error rates were shown to be on the order of 1% and thus $0.01 * 2000 = 20$, and likewise across all strand designs studied for the `Alignment Matrix` algorithm error rates ranged on the order of magnitudes of $[0.001 - 0.01]$ for a sample size of 10^4 and thus also satisfies the threshold.

Thus, by linearity of expectation, both P_l and $\overline{P}_B$ of Equations 2 and 3 are normally distributed with means that are averages of the true means of the $\mathbf{I}_{l,s}$ Bernoulli random variables. To calculate the variance of $P_{l,s}$ we use $\boldsymbol{Var}(\mathbf{I}_{l,s})/|R|_s$ which we estimate as follows:

$$\boldsymbol{Var}(P_{l,s}) = \frac{P_{l,s}(1 - P_{l,s})}{|R|_s} \tag{4}$$

Then we can calculate the variance of P_l as:

$$\boldsymbol{Var}(P_l) = \frac{1}{|S|^2} \sum_{s=0}^{|S|} \boldsymbol{Var}(P_{l,s}) \tag{5}$$

Here we assume that byte errors are independent across strands. In the case of $\overline{P}_B$ this is not the case as we have seen that byte error rates *within* a strand for the `Alignment Matrix` and *HEDGES* decoding algorithms are not independent of previous bytes. That is, if the error rate increases for $P_{l,s}$ we expect $P_{l+1,s}$ to increase as well. Therefore, we include covariance terms between byte positions in the calculation of $\boldsymbol{Var}(\overline{P}_B)$ as follows:

$$\boldsymbol{Var}(\overline{P}_B) = \frac{1}{|L|^2|S|^2} \left(\sum_{l=0}^{|L|} \sum_{s=0}^{|S|} \boldsymbol{Var}(P_{l,s}) + \sum_{s=0}^{|S|} \sum_{i<j}^{|L|} 2\boldsymbol{Cov}(P_{i,s}, P_{j,s}) \right) \tag{6}$$

The value of $\boldsymbol{Cov}(P_{i,s}, P_{j,s})$ is the covariance of sample means of the Bernoulli random variables of $\mathbf{I}_{i,s}$ and $\mathbf{I}_{j,s}$, which can be written as:

$$\begin{aligned} \boldsymbol{Cov}(P_{i,s}, P_{j,s}) &= \boldsymbol{Cov}\left(\frac{\sum_{r_i=0}^{|R|_s} \mathbf{I}_{i,s,r_i}}{|R|_s}, \frac{\sum_{r_j=0}^{|R|_s} \mathbf{I}_{j,s,r_j}}{|R|_s} \right) \\ &= \frac{1}{|R|_s^2} \sum_{r_i=0}^{|R|_s} \sum_{r_j=0}^{|R|_s} \boldsymbol{Cov}(\mathbf{I}_{i,s,r_i}, \mathbf{I}_{j,s,r_j}) = \frac{1}{|R|_s} \boldsymbol{Cov}(\mathbf{I}_{i,s}, \mathbf{I}_{j,s}) \end{aligned} \tag{7}$$

Here we have used the bilinear property of covariance to obtain an equation for the covariance of the measured byte error rates in terms of the covariance of the Bernoulli random variables which we can estimate using the following relationship between expected values and covariance:

$$\begin{aligned} \boldsymbol{Cov}(\mathbf{I}_{i,s}, \mathbf{I}_{j,s}) &= \boldsymbol{E}[\mathbf{I}_{i,s}\mathbf{I}_{j,s}] - \boldsymbol{E}[\mathbf{I}_{i,s}]\boldsymbol{E}[\mathbf{I}_{j,s}] \\ &= P(\mathbf{I}_{i,s} = 1)P(\mathbf{I}_{j,s} = 1|\mathbf{I}_{i,s} = 1) - P(\mathbf{I}_{i,s} = 1)P(\mathbf{I}_{j,s} = 1) \end{aligned} \tag{8}$$

The value of $P(\mathbf{I}_{i,s} = 1)$ is the probability of a byte error rate at position i in strand s, which we can estimate with $P_{i,s}$. The conditional probability $P(\mathbf{I}_{j,s} = 1|\mathbf{I}_{i,s} = 1)$ captures the probability that a byte will be in error given the preceding byte registered an error ($j > i$). We do not measure this conditional probability directly, but a conservative estimate of the covariance and the following overall variance can be made by assuming $P(\mathbf{I}_{j,s} = 1|\mathbf{I}_{i,s} = 1) = 1$ which will result in a covariance that is larger than the true value. Finally, we arrive at our estimation of covariance $\boldsymbol{Cov}(\mathbf{I}_{i,s}, \mathbf{I}_{j,s}) = P_{i,s} - P_{i,s}P_{j,s}$. With a calculation for the variances of each average error rate used, we use each mean ± 1.96 standard deviations to calculate our error bars.

A.5. Calculating Overall System Density

We determine the total density of a storage system as the product of four information rates as follows $\phi_T = 2\phi_{\mathrm{RS}}\phi_{\mathrm{overhead}}\phi_{\mathrm{HEDGES}}$. Here the factor of 2 represents the maximum capacity of information that can theoretically be stored per base, ϕ_{RS}

represents the rate of the Reed-Solomon code used to withstand errors that persist after decoding the inner code, ϕ_{overhead} represents the ratio of information carrying bases to the total number of bases that need to be stored when implementing the strands needed for the storage system, and ϕ_{HEDGES} is the rate of the inner code used to translate digital information into bases that construct the molecules of the DNA storage system. While other terms require some calculation, the ϕ_{HEDGES} term is simply the rates that were chosen as parameters for this study.

The value of $\phi_{overhead}$ needs to consider strand length, the amount of that strand which will carry information corresponding to the raw information to be stored, and the different portions of the strand that will be needed to effectively recover information from the storage system. Implementing a DNA storage system typically requires an indexing region on each strand to allow for the information that is retrieved from unorganized sequencing data to be placed in the proper order. Furthermore, functional bases may be required within a strand to enable functionality such as PCR, transcription, etc. We calculate $\phi_{overhead}$ with the following formula for a molecule of L bases:

$$\phi_{\text{overhead}} = \frac{L - \frac{8B_{\text{index}}}{2\phi_{\text{HEDGES}}} - L_{\text{functional}}}{L} \tag{9}$$

There are several parameters that need to be chosen in Equation 9. B_{index} represents the number of bytes that are used for indexing strands. In this work we choose $B_{\text{index}} = 4\text{bytes}$ which allows for more than 4 billion molecules to represent some amount of data. Even for molecules of just 100 bp, 4 billion molecules surpasses the total base output of high performance flow cells such as PromethION Flow Cells. While this amount of indexed molecules may limit the amount of data that can be indexed together, it has been shown that physically addressing molecules through PCR (Organick et al. (2018)) and multi-stepped PCR (Tomek et al. (2019)) is a viable solution to increase the address space of a single pool of molecules by thousands (Organick et al. (2018)) to potentially millions (Tomek et al. (2019)) of additional levels of addressing. The bases associated with this addressing is modeled using the $L_{\text{functional}}$ term which we set to 137 bp, the number of bp that are used as buffers/promoters in our strand designs. Given that the primers used in the aforementioned molecule addressing strategies are approximately 20 bp, $L_{\text{functional}} = 137\text{bp}$ offers a sufficient budget for multiple primer addresses in the assumption that the buffer/promoter regions are simply repurposed.

Calculating ϕ_{RS} requires finding the minimum Reed-Solomon parity required to reliably recover a set of data. In this work we assume RS codes over GF(2^8), and thus codewords of length $255 = |D| + |P|$ with $|D|$ data symbols (bytes) and $|P|$ parity symbols. Once a sufficient $|P|$ is determined, the density of the RS outer code becomes $\phi_{\text{RS}} = \frac{|D|}{255}$.

To come to a conclusion on what Reed Solomon code design should be chosen, we use $\overline{P}_B$ to calculate the probabilities of decoding success of a single codeword and then project that probability to a larger storage system. This is done with Equation 10. This equation sums up the probability of events that will cause a RS codeword to fail decoding, e.g. $t > \lfloor \frac{P}{2} \rfloor$ for t corruption errors in a RS codeword consisting of $255 = D + P$ total symbols with P redundant symbols and D data symbols. Considering each RS codeword must decode successfully, and assuming each is independent of each other, the mean time to failure (MTTF) will be $\frac{1}{1-(P_{RS})^{T/D}}$.

$$P_{RS} = 1 - \sum_{k=0}^{255-\lfloor \frac{P}{2} \rfloor -1} \binom{255}{k} (1 - \overline{P}_B)^k (\overline{P}_B)^{255-k} \tag{10}$$

With each individual rate component known, the following equation can be used to combine them to determine an overall bits/base density of a storage system:

$$\phi = 2\phi_{\text{HEDGES}}\phi_{\text{RS}}\phi_{\text{overhead}} \tag{11}$$

A.6. CTC Models

For DNA reads, **Beam Trellis** and **Alignment Matrix** algorithms are integrated with Oxford Nanopore Technology's open source basecaller Bonito (https://github.com/nanoporetech/bonito). Our implementations extend this code base starting from commit `d36dfca`. We use the CTC architecture provided with Bonito, with model version `dna_r9.4.1@v2`. For RNA reads, we use open source basecaller and model RODAN retrieved from (https://github.com/biodlab/RODAN), and our implementation is based on commit `029f7d5`.

A.7. Preprocessing FASTQ Reads

In our analysis, we post-process the raw sequencing reads from nanopore devices in order to identify which sequencing read belongs to each encoded strand, and to remove low quality reads that are unlikely to provide useful information regarding byte/base error rates. Our process is outline in Figure 2. We start with the FASTQ files of nanopore reads provided by the ONT basecallers, and pass these through FrameD's sequencing read mapping utilities Volkel et al. (2023). The output of this step provides a map that links sequencing read IDs with the indexes of the encoded strands. We then take this mapping information and create a random subset of reads for each individually encoded strand. These subsets are used to partition the original FASTQ file and the raw electrical data that is stored in the FAST5 format to create the set of reads that are used for the decoding analysis that results in byte/base error profiles.

During the initial analysis of the ONT basecall data to create the index/read ID map, we pass each read through a series of filters. The first filter we apply is an alignment filter between the sequencing read and the $3'/5'$ buffer regions that we apply to the ends of each encoded strand. The buffer regions are intended to provide protection from skipped bases that may occur on the transient entrance/exit of a molecule traversing the nanopore, but we also require their removal prior to decoding by the HEDGES algorithm. This is because HEDGES will not recognize the additional buffer regions as part of the overall message, and so decoding will likely fail. In order to ensure that the alignment is a reasonable quality, we require that each alignment has at least 70% matching bases within the alignment. Following the alignment, the region of the sequencing read that is aligned to is removed and the subsequent remaining read's length is inspected. If the read is either too long, or too short, we remove it from consideration with the reasoning that extremely short reads indicate incomplete reads that are highly unlikely to decode and long reads indicate chimeric read events. We avoid including these short or long reads with the goal of avoiding anomalous reads in the inclusion of base/byte error analysis so that typical nanopore behaviour that results in decoding failures can be reasoned about. For the lower bound, we

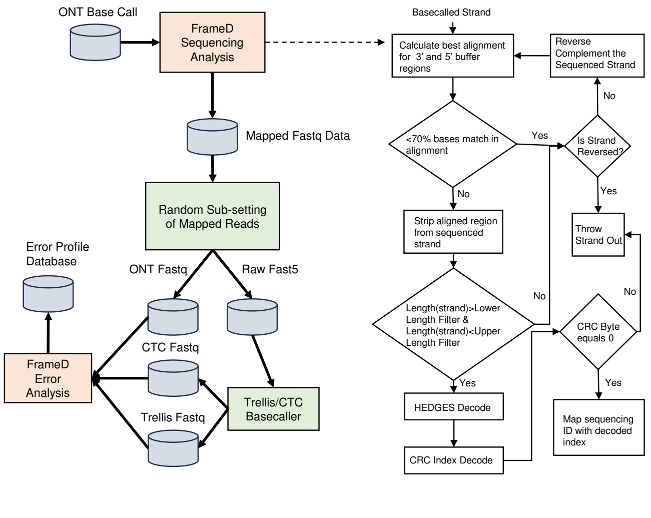

Fig. 2: Diagram of our workflow for determining a working set of nanopore reads to evaluate against our decoding algorithms.

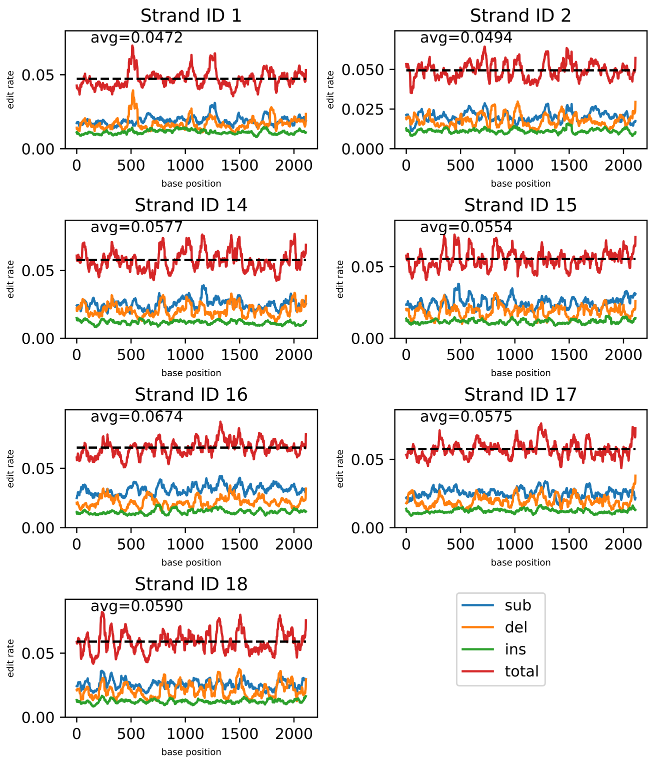

Fig. 3: Basecalling error rates measured for each strand of the 0.167 HEDGES rate design of length 2297 bp over a sample of 100k reads for each strand. Error rates are measured over the 2160 bp that are carrying the encoded information. Each line represents a rolling arithmetic mean for errors over 50 bp windows for substitutions (sub), deletions (del), insertions (ins), and the total number of errors. Dashed horizontal line represents the average error rate over the entire strand calculated by summing all errors at all positions and dividing by the total number of reads and positions.

use a value that is 6-7% shorter relative to the length of the region that encodes information, and for the upper bound we use a value of 300-400 bases longer relative to the length of the region of the molecule that encodes information. As the final step, we use the HEDGES algorithm to decode the index of the read, and as a final check to ensure that the index bytes are decoded correctly we calculate a CRC check byte to avoid incorrect indexes being attributed to sequencing reads.

A.7.1. Basecalling Error Rates

As supporting evidence for our sequencing post-processing methodology, we calculate the edit-rate for each strand of the 0.167 HEDGES rate as shown in Figure 3. We calculate the edit rate by determining the edit operations used to transform the originally encoded strand to the read observed during sequencing after removing buffers (e.g. only HEDGES encoded bases). By not including buffers, we are able to directly see what error rates the error correction code is being faced with. We then accumulate the number of edits and type at each base position to determine a base error rate. During analysis we found that short reads manifest with high numbers of deletion errors towards the terminal base positions of the encoded strand. To account for noise in the edit rates, we average over a rolling window of 50 bp to allow for local variation and global trends across reads to be observed. From this data we can concluded that deletion error rates (and all other rates as well) remain consistent across the strand. This indicates that there is not a large positional influence in error rate across strand positions due to sequenced molecule length.

A.7.2. Q Score of Partitioned Reads

To further investigate the impact of our filtering on the quality of reads kept for analysis we study the impact on the distribution of quality scores determined by the ONT basecallers. Figure 5 shows quality score distributions for reads covering all density and length design points covered in this work. The line annotated as *Total* represents the quality score distribution for every read in the sequencing run, other lines are labeled with encoded strand identifiers and these distributions represent the distributions for each read *after* they have passed through the filtering steps of Figure 2. We also label the plots with two means of each distribution. The first mean is the mean of the entire distribution, and the second mean represents the mean of the distribution if all Q scores less than 9 are ignored. The cutoff point of 9 was chosen based on ONT's default setting of determining poor quality reads. Based on the average of the entire distribution, the filtered reads generally have an average that is larger than the total population. However, it is apparent that this shift is a result of a large number low quality reads between Q score of 5 and 6 that do not exist in the filtered read sets. If we consider a Q score cutoff of 9, we can see that the averages of the total distribution and the filtered sets are considerably closer. Furthermore, we can also observe that the peaks of the filtered distributions do not bias towards the high Q score tail of the total distribution, indicating that our filtering process does not have a preference for very high quality reads. Provided that our filtering keeps reads close to the desired read length, we can infer that the low quality score reads lost in our filtering correspond to short-length reads and further motivates eliminating short reads from consideration.

There was some cross-batch variation in quality scores. For example, the average quality score for 0.5 rate HEDGES was

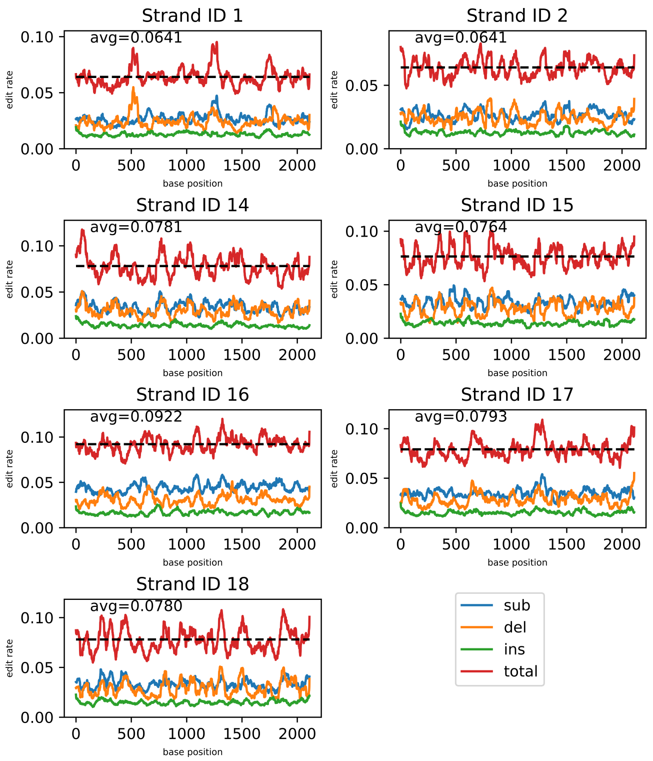

Fig. 4: Rolling average base edit rates over a 50 bp window for the open source Bonito-CTC basecaller applied to the 0.167 HEDGES library across 100k reads.

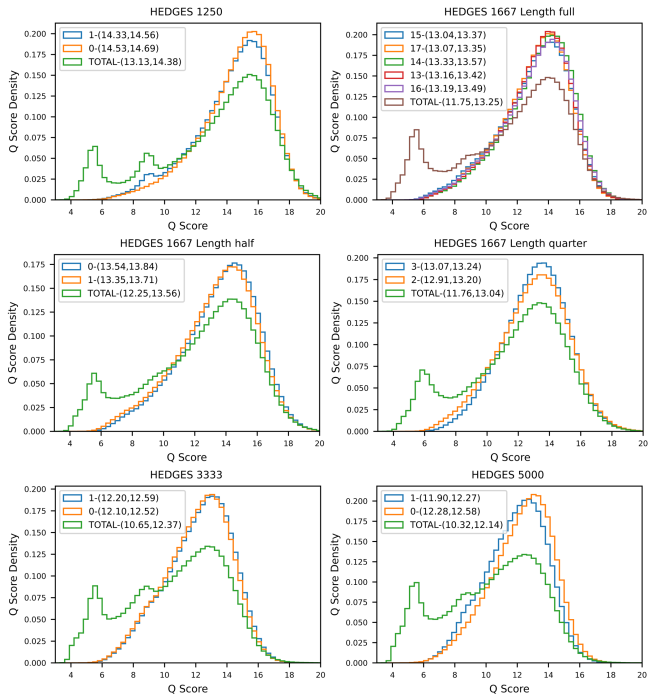

Fig. 5: Q score distribution for filtered and all reads for sequencing runs performed for each strand design.

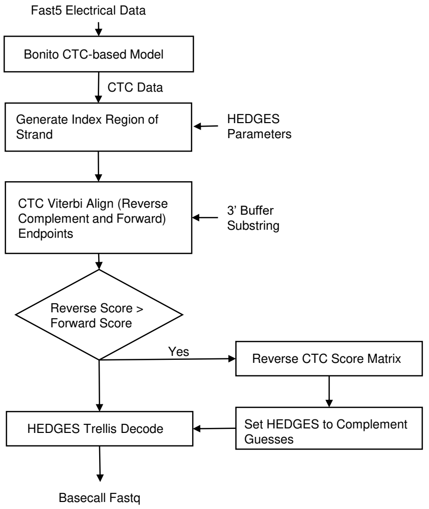

Fig. 6: Processing flow of electrical data stored in FAST5 files throughout the process of decoding HEDGES encoded strands using both the **`Alignment Matrix`** and **`Beam Trellis`** decoders.

significantly lower (10.32) than that of the 0.125 rate (13.13). While the encoding densities are different, the constraints imposed on the encoder are the same, e.g. we only allow for homopolymers of length 3 at most, a fixed GC content to be 50%, encoded length ≈ 2160. Also, we observe that there is no significant change in Q score across the 5 different strands of the *HEDGES 1667 Length full* run. Thus, we attribute the quality score variation to variations in the nanopore flow cells.

A.8. Preprocessing CTC Matrices

To utilize the decoding algorithms that leverage CTC scores for decoding HEDGES codes, there are several steps that need to be completed beforehand. Figure 6 illustrates the preprocessing required to transform electrical data into the proper CTC form for our studied algorithms. First, the FAST5 data needs to pass through a CTC-based machine learning (ML) model. In the case of DNA molecules, this model is an open source CTC version of the Bonito basecaller, for RNA strands the model is the open source RODAN ML model Neumann et al. (2022). These models generate the CTC matrices that are required to decode each nanopore read.

As mentioned, there are bases which serve molecular function purposes that surround the payload that we are interested in studying the decode error rates of. Thus, the CTC matrices provided by the ML models will also include scores related to these bases that are unrelated to the encoded payload. The CTC scores that correspond to the auxiliary bases are pruned so that the algorithms can make higher quality alignments for messages without noise introduced from alignments to non-data regions. Supplemental Figure 6 describes our workflow for pruning CTC information and understanding the read direction that the CTC information represents. The CTC pruning process performs alignment in both directions, forwards and reverse complement. This is especially important for DNA reads as any read can be forward or reverse complement, and we need to know which bases to generate in to properly decode relative to the CTC data. However, for RNA we only need to consider alignment in the

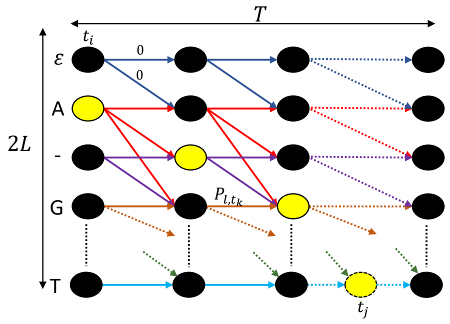

Fig. 7: Trellis used in the process of aligning buffer regions using CTC scores for the purpose of determining start points for the HEDGES decoders.

forward direction since it is only a single strand molecule. Once alignment is complete, if the reverse score was found to be larger we reverse the direction of the CTC matrix and set the HEDGES base-generation to emit complements. We finally are now able to perform trellis decoding for the HEDGES strands using CTC scores during which we generate a FASTQ basecall file with the final decoded message.

The process of aligning a known base sequence to a CTC matrix is a common strategy referred to as *forced alignment* that has been applied in speech recognition to determine regions of the CTC scores that correspond to utterances. In our case, the utterances will be the buffer/index regions of our DNA/RNA strands, and so we follow a procedure similar to that provided by Kürzinger et al. (2020), which is outlined in Figure 7. The algorithm is set up as a trellis, with each state corresponding to a base in the known sequence, or a blank that is inserted between each base. An initial state ϵ is included to allow for alignments to be found at any starting time point in the CTC matrix. The trellis states are evaluated for each time step in the CTC matrix, with edges between states that correspond to valid CTC encodings of the know base sequence. For example, if the known base sequence has a repeat such as GG then there cannot be a direct edge between each of the G states in the trellis. There must be blank symbol in between. This is not necessary for non-repeats as shown in Figure 7 with the edges connecting A to G.

The score of each state is calculated as the maximum of the score of all incoming states summed with the log-probability of the state's symbol at the given time point. The following equation provides the score $P_{l,t}$ for state l at time t.

$$P_{l,t} = \begin{cases} \max(\mathrm{CTC}[l,t] + P_{l,t-1}, \\ \mathrm{CTC}[l,t] + P_{l-1,t-1}, \\ \mathrm{CTC}[l,t] + P_{l-2,t-1}) & \text{if } l \neq l-2 \\ \max(\mathrm{CTC}[l,t] + P_{l,t-1}, \\ \mathrm{CTC}[l,t] + P_{l-1,t-1}) & \text{if } l = l-2 \end{cases} \quad (12)$$

The first case of Equation 12 is for the case of non-repeats between two consecutive bases in the sequence, and the second is for the repeat case. With this definition for scoring, the trellis can be filled out so that each state is assigned a back-trace path corresponding to the edge taking during the maximum of Equation 12. After this is done, the starting point for the back trace pass is taken as $t_j = \arg\max_i(P_{2L-1,i})$, the time step that results in the maximum score for the final base in the known sequence. Starting from here we back trace until the first base in the sequence is reached, e.g. following the highlighted states until t_i in Figure 12. Thus, the time steps from t_i to t_j are taken as our alignment.

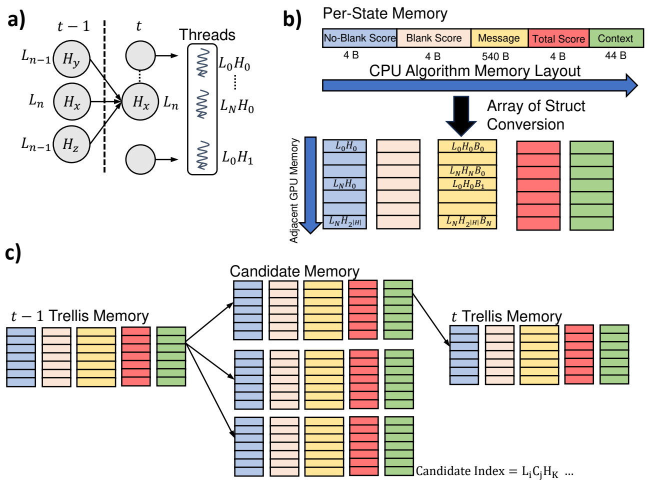

Fig. 8: **a)** Trellis to thread mapping used for constructing parallel work units for the `Beam Trellis` algorithm. **b)** Memory layout of data related to each state at trellis time step $t-1$. **c)** Layout of temporary candidate data that is used to determine the best incoming path to choose for a given state.

A.9. GPU Implementations

A.9.1. GPU Beam Trellis Port

We provide a general outline here of our GPU port of the work by Chandak *et al.* Given that GPU implementations of algorithms are highly dependent on several design choices such as thread layout, memory layout, and resource usage such as local shared memory spaces, we aim to show that the choices that were made for the GPU implementation of the `Beam Trellis` algorithm are reasonable and should not hinder the algorithms performance relative to our `Alignment Matrix` algorithm. An outline of our design choices is presented in Figure 8. Our implementations are based on the NVIDIA CUDA GPU programming platform.

The first step we take in constructing our GPU implementation for the `Beam Trellis` algorithm is choosing the parallel units of work and their layout on the GPU device. For trellis-based algorithms, the computation for each state's score at a given step are all independent of each other. Also, given that there are a large number of states for the `Beam Trellis` algorithm, $\approx 2000 \times 256 = 512000$, parallelization over the states will easily keep a GPU device busy. Thus, as shown in Figure 8 we assign a thread to each state, and we order each thread co-lexicographically over the number of length states L and convolutional states H. That is, for a strand length of N and number of convolutional states M, thread indexes are ordered according to $L_0H_0, L_1H_0, ..., L_0H_1, ...L_{N-1}H_{M-1}$. This ordering becomes important when considering memory access patterns of threads and maximizing the amount of read coalescing that can impact memory throughput of the implementation.

The second step for our GPU implementation was to reconfigure the memory layout of data structures to enable GPU memory access coalescing. The core data structure that we associate with each state is shown in Figure 8b . This includes 5 different fields: ***No-Blank Score*** a 4-byte float that stores the aggregated scores of all paths to a given state that do not end in a blank, ***Blank Score*** a 4-byte float that stores the aggregated scores of all paths to a given state that do end in a blank, ***Message*** a 540-byte bit-array that stores the base-message using 2 bits per base that corresponds to a given state for a given trellis-step, ***Total Score*** a 4-byte float aggregating non-blank and blank scores, and ***Context*** a 44-byte object that is responsible for keeping track of which base to emit based on tracked constraints such as GC balance and homo-polymer runs. Typically, for a CPU implementation, these fields should be adjacent when evaluating each state because there will be high temporal locality between the fields. However, because GPU threads all execute identical instructions in lock-step given their single instruction multiple thread (SIMT) execution model, a GPU device's memory throughput is better optimized when each thread accesses adjacent memory locations. Thus, similar to how we order threads, memory locations are co-lexicographically ordered over L_iH_j. Note, because the message field is so large (540-bytes) and thus will contribute to a large portion of the memory bandwidth, we further re-arrange the bit-array to be co-lexicographically ordered over $L_iH_jB_k$ where B_k indicates a bit position in the bit vector.

To see why arranging threads and memory in this manner results in adjacent threads accessing adjacent memory we can analyze the memory access patterns for each thread with this given memory layout. We co-locate threads that have a common convolutional state H_x. For example, $L_0H_x, L_1H_x, ..., L_{N-1}H_x$ are adjacent. Each thread in this set will need to access the memory corresponding to three separate candidates, each corresponding to three different valid previous convolutional states (Figure 8a). Given that H_x is constant amongst these threads, they will all have the same incoming previous states. Furthermore, for some thread L_nH_x in this set, the incoming candidates will have position portions of either L_{n-1} or L_n. For L_{n-1} this means that the memory access according to each thread will be $\varnothing, L_0H_y, ...L_{N-1}H_y$, which is a favorable access pattern for coalescing given the memory layout. The access $\varnothing$ is used for the case that there is no L_{n-1} candidate for L_0. For L_n, the state H_x does not change, nor the position, thus the access pattern becomes simply $L_0H_x, L_1H_x, ..., L_{N-1}H_x$.

The memory accesses covered so far only concern with reading memory associated with incoming states of the previous trellis time step. Another memory region of concern is the memory used to store candidate results (Figure 8c). This memory holds the same memory fields as shown in Figure 8 b), but three copies are needed for each thread to account for each of the three possible incoming candidates. For this candidate memory, we lay it out such that the co-lexicographic order is based on $L_iC_jH_k...$. This enables memory accesses to be coalesced since each state will fill in their candidates in the same order as every other adjacent state. That is, candidate coordinates of $L_0C_0H_x$ and $L_1C_0H_x$ will be written to at the same time during SIMT execution. We could also change the coordinates of candidate memory to be $L_iH_jC_k...$, but because our implementation fixes each block and thus warp to have a constant H_j this reordering would be inconsequential to memory coalescing.

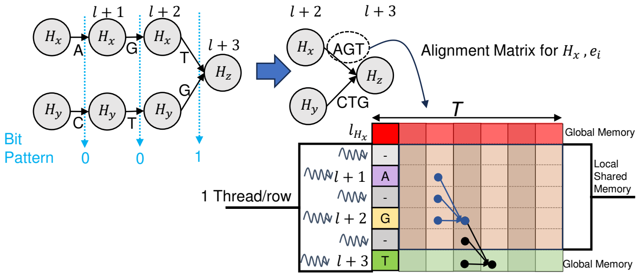

Fig. 9: Outline of GPU implementation for the `Alignment Matrix` algorithm.

A.9.2. Alignment Matrix

The outline of the key optimizations done for the `Alignment Matrix` implementation is shown in Figure 9. First, we noticed that for the HEDGES algorithm there is a pattern where the transitions between states are self-transitions when the bits conveyed by a base are 0. That is, the state does not change when no new information is transmitted by a base. For this reason, we do not need to calculate edge scores as it is trivial to determine the best transition coming into a state for trellis steps $l+1$ and $l+2$ as indicated by Figure 9. Once a transition is reached between two trellis steps that require score comparisons to determine the maximum path ($l+2$ and $l+3$), we calculate the alignment score for the newly added base along with all bases accumulated along the 0 bit pattern paths. For example, the new base added on the transition between state H_x at step $l+2$ going to H_z at step $l+3$ is T, but the actual bases utilized in the alignment matrix calculation is AGT.

With the bases known on each transition, we can then calculate a score to compare two incoming edges reaching a state. This results in each edge requiring a calculation of its alignment matrix which is formed as shown in Figure 9. The top row of the matrix is a length T array of scores which corresponds to the alignment scores of the best path for state H_x at step l. These values are stored in global memory since they need to be utilized by succeeding steps in the trellis. Likewise, the final row for the last base needs to be stored in global memory since this row needs to be propagated along the trellis to facilitate future edge scores. However, there are a number of alignment scores that can be stored in the GPU's shared memory since they are intermediate values that are not necessary for future calculations. These are the rows of the alignment matrix that correspond to bases that are not the last base in the edge's complete string, or rows that correspond to blanks represented by - in Figure 9. This reduces the amount of global memory bandwidth, and also ensures that the required memory from the GPU is only proportional to T and not $L \times T$ which would be necessary if the entire alignment matrix was naively propagated through the trellis. Thus, the matrix alignment algorithm can maintain memory linear in strand length instead of quadratic since $T = O(L)$.

Data flows in the alignment matrix from the first column to the last column, with each column corresponding to a time step which consumes the previous time step's data as shown by the connection of matrix elements in Figure 9. Unfortunately, that means that calculating elements in successive columns needs to be serialized. However, this pattern also means that all rows in the same column can be calculated independently. This leads us to assign threads

to each row for a given alignment matrix calculation. Thus, the number of threads launched per trellis step is $H \times E \times L'$ where E is the number of edges on a transition. Typically, $E = 2$ when 1 bit is transmitted per base, but provided that a base can represent up to 2 bits $E = 4$ is also possible for different HEDGES code rates which is supported by our implementation. L' here is the number of independent rows that are being calculated for a transition edge. This depends on the HEDGES rate as well since lower code rates will increase the number of 0's in the bit pattern which increases the number of rows calculated at each step. For a fixed strand length, L' will impact run time such that lower density codes will decode faster than more dense ones. The reason is that more dense codes will not be able to leverage shared memory as much and require more global memory transfers.

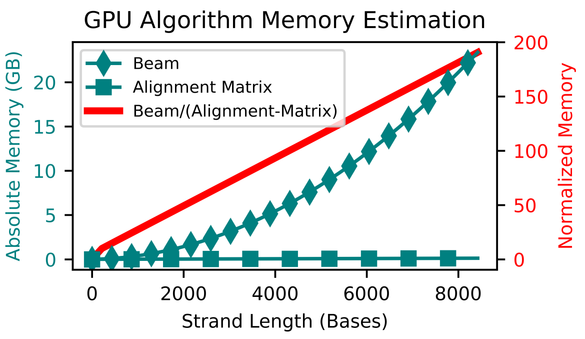

Fig. 10: Average memory consumption comparisons between the `Beam Trellis` and `Alignment Matrix` algorithms.

A.10. Soft Decoder Memory Comparisons

Based on both of our soft decoder implementations, we outline the calculations made to determine their memory consumption. Starting with the `Beam Trellis` algorithm, we calculate the total memory required with the following equation, assuming N for the total number of positions and M total convolutional states.

$$\text{Beam Trellis Memory (Bytes)} = 5 \cdot M \cdot N \cdot (56 + \frac{N \cdot 2}{8}) \quad (13)$$

As we showed in Figure 8 b), each state requires 56 bytes plus the memory required for the message. Because the 66 bytes is constant with message size, we represent the message memory separately in Equation 13 as $\frac{2 \cdot N}{8}$ where we use 2 bits per base of a message. This quantity is multiplied by $M \cdot N$ because every state needs its own version, and another factor of 5 is included to account for 3 candidate copies and 2 copies required to store trellis time step $t-1$ and time step t (Figure 8 c)). Using this equation we are able to project an estimate for memory as the number of states changes or the length of the strand changes.

The memory of the `Alignment Matrix` algorithm is allocated over a small set of critical arrays that are required to track alignment information, paths through the trellis, and context required by the HEDGES algorithm. The arrays we use are: a ***Context*** array of dimension $2 \times M$ 44-byte data structure used to manage the HEDGES state between steps in the trellis, a $T \times M$ array of 4-byte floats named the ***Forward*** array that holds the alignment results across the all T steps of the CTC matrix for each of the M convolutional states (first row of the array highlighted red in Figure 9), a $T \times M \times 2$ array of 4-byte floats named the ***Forward-Edges*** that tracks the alignment for each edge during a trellis transition (last row of the array highlighted green in Figure 9), and we utilize two $M \times N$ arrays of 8-byte values where one serves as the back-trace array after the trellis is complete and the other stores the bases that are emitted along the best-path edges. This leads to the following summation of memory for the `Alignment Matrix` algorithm as a function of the number of convolutional states and strand length.

$$\text{Alignment Matrix Memory (Bytes)} = M(12T + 16N + 88) \quad (14)$$

From Equations 13 and 14, it is clear that the `Beam Trellis` algorithm's memory consumption is quadratic in strand length, while the memory consumption of the `Alignment Matrix` algorithm is only linear in strand length. Note, T, the dimension of the CTC matrix is generally not constant for each read even for the same strand length L. Thus, we require some assumptions for T in order to calculate the bytes required for the `Alignment Matrix` algorithm. We choose T to be the average CTC time dimension measured over the 400 reads used for benchmarking, which we measure to be 7400 as indicated in Figure 11 which displays the distribution of T values over these 400 reads. Given this assumption, we project to different strand lengths by scaling the T and N terms of Equations 13 and 14 by a factor of $\frac{N'}{N}$ for some new strand length of N' bases. For our memory calculations, we fix $N = 2160$ bases and $M = 256$ convolutional states. Results for projecting memory consumption at different strand lengths is shown in Figure 10.

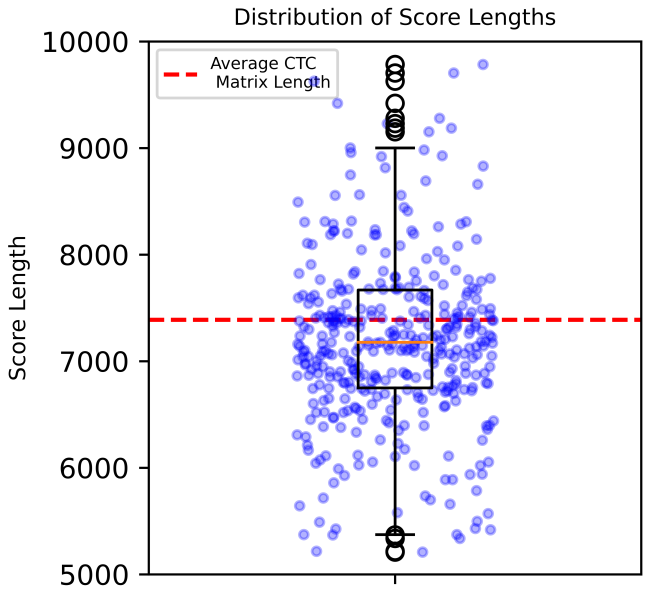

Fig. 11: Distribution of CTC matrix time lengths for 400 reads that were analyzed in detail for run time and memory benchmarking.

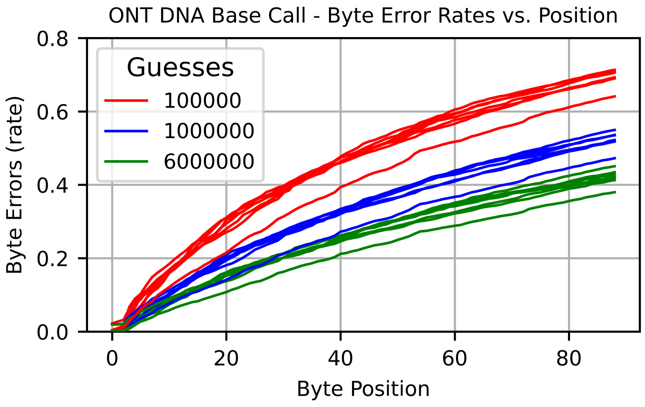

Fig. 12: Byte error rate as a function of byte position when applying the HEDGES Tree search algorithm to ONT basecaller outputs for 3 different guess budgets. All curves correspond to the 1/6 encoding rate strands.

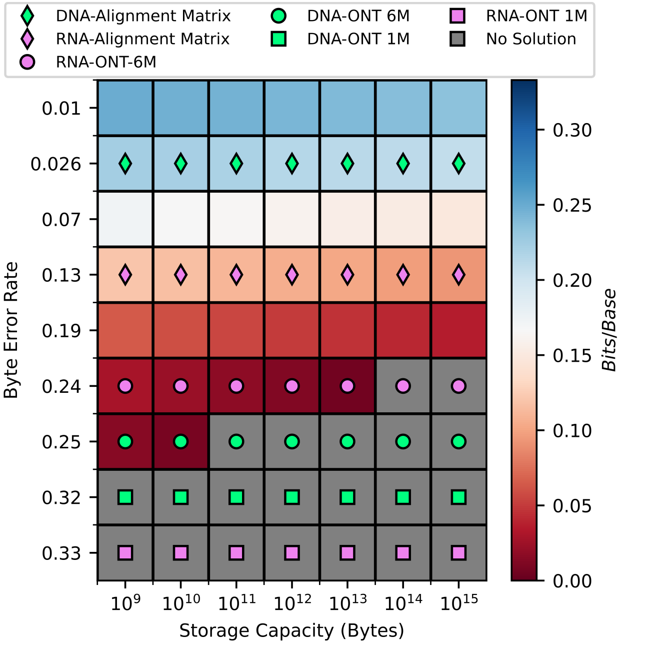

Fig. 13: Byte error rate (rows) and storage capacity (columns) versus the achievable density (color bar) when assuming the 1/6 HEDGES rate code. Rows labeled with a marker indicate a byte error rate that was measured for that decoder. All DNA markers pertain to error rates measured on the full 7 strand library for the 1/6 rate HEDGES encoding. RNA/DNA-ONT XM refers to decoding RNA/DNA basecalls using X million guesses.

B. Additional Hard Decoding Analysis

Figure 12 shows the relationship between byte error rate versus position for each encoded strand when the ONT basecallers are decoded with the HEDGES hard decoding algorithm. This data confirms the behaviour observed by Press et al. (2020) of the Tree decoding algorithm where as position increases along the length of the strand the probability that the byte can be decoded decreases. The reason this occurs is due to running out of guesses before the read can be completely decoded. Increasing the number of guesses can decrease the byte error rate at each position, but the correlation between position and error rate still remains. We also note here that there is generally not a significant difference between each encoded strand's byte error rate curves, or locations within each curve that have sharp changes in byte error rate. This indicates that events from the ONT basecaller that causes HEDGES to fail are not strongly correlated to any given position.

To show how byte error rates impact the cost for a given amount of reliability and storage system of a certain size, we plot a density heat map for various byte error rates and system sizes in Figure 13. In this analysis we assume a MTTF $= 10^6$ in all cases. Based on this data, we show that the byte error rates associated with using the HEDGES tree based decoder result in no solution, or very low densities, when storage systems are larger than 1 TB. A scenario in which no solution occurs means that we exhausted all Reed-Solomon code designs in terms of the amount of the codeword allocated to data and parity symbols before the MTTF threshold was surpassed. Furthermore, even for small storage systems of 1 GB, densities are $<$ 0.5 bits/bp for this decoder. These low densities confirm that the baseline hard decoder is not suitable for a single-read storage system in a nanopore sequencing setting.

C. Strand Length Analysis

Given that the shorter strands have been shown to have lower byte error rates, we are interested in understanding the error rate relationship as a function of byte position so that we may develop a method to project how strand length may impact other HEDGES densities. Figures 14 and 15 show how the byte error rate trends in relation to position when averaging over each individual strand and when considering strands individually respectively. Figure 15 shows that the shorter length strands have byte error rates that are similar on average to that of the full length strand at each respective position. We do note that the average byte error rate curves for the half and quarter length strands lie above and below that of the full length strand respectively. However, given that we have normalized the samples to contain reads between values of 15.1-15.4 of Q scores for both figures to factor out flow cell variation, we consider the deviation of the shorter strand byte error rates to be a product of how the model and decoder respond to different base sequences. Supporting this idea is Figure 14 which shows that there are full length strands that have individual byte error rate profiles that are close or that their error bars encompass the byte error rate of the individual short strands. We conclude from this data that an estimation of an error rate profile for the other studied HEDGES rates at shorter strand lengths can be derived by truncating their respective byte error rate curves appropriately.

D. Q Score Analysis

To analyze the impact of quality of read provided to the decoder, we construct 15 bins of range 0.3 between Q Scores of 10.9 and 15.1 (Supplemental Figure 16). For each bin, and for all reads of each strand that fall into each bin, we select a 10k random subset for a total of 2.55 million reads. Each point represents $\overline{P}_B$ calculated over all positions and strands for a given design.

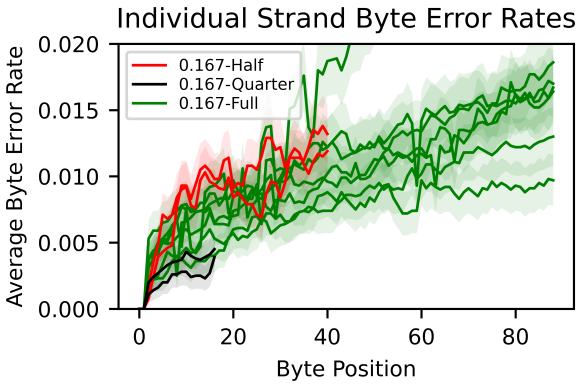

Fig. 14: Byte error rate vs position for each strand across the three different synthesized lengths for the 1/6 encoding rate. Half, Quarter, and Full each refer to the three different strand length designs in increasing order. Error bars that represent a 95% confidence interval are calculated for each byte based on the variance calculation of Supplemental Equation 4.

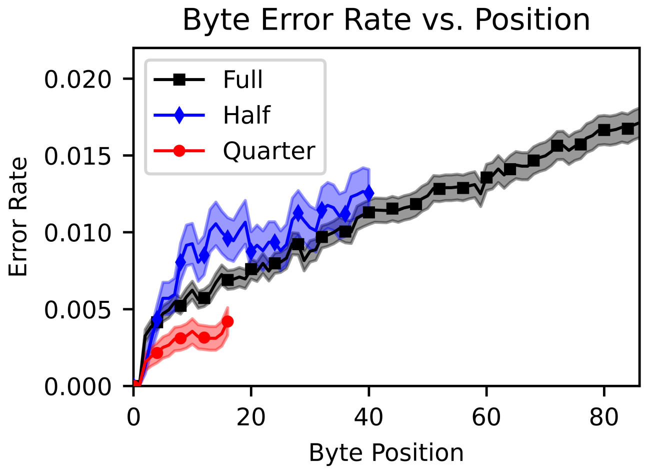

Fig. 15: Byte error rate for each byte position average across all synthesized DNA strands for the 3 different lengths studied for the 1/6 rate HEDGES encoding. Error bars are calculated according to Equation 5 to represent a 95% confidence interval.

E. Average Decode Latency Analysis

Figure 17 plots the relationship of the decode time as a function of strand length (including buffer regions). This plot provides information on the total decode time (left) and for two key steps in decoding, CTC matrix generation (top right), and the `Alignment Matrix` algorithm (bottom right). With a quadratic fit, we can clearly see a relationship between strand length and decode time for both the overall decoding time as well as the `Alignment Matrix` algorithm step as would be predicted from the algorithm's time complexity. From this data we can also conclude that there is a linear relationship CTC matrix generation and the length of a strand, which should be expected as well given that the number of samples that will need to be processed by the ML model will scale linearly with strand length. Furthermore, the model's CTC generation is approximately an order of magnitude faster than decoding the matrix which is the reason why that the overall time closely follows the time of the Alignment Matrix Algorithm.

Based on these timing results we can consider some tradeoffs when considering more or less computationally intense models used for CTC matrix generation. When considering a model that has a longer inference time due to a larger number of parameters we would expect overall a higher accuracy for both hard and soft decoding. However, given that the byte error rate for our Alignment Matrix algorithm is already at 2-3% for the 1/6 code rate, the increased accuracy from such a large model is unlikely worth the increased time for inference. This is because the current high accuracy model used in our experiments result in 65% of the encoding capacity of 0.33 bits/base than can be reached for the 1/6 encoding rate design. Furthermore, we envision a real-time system in the long run that can employ high accuracy decoding techniques like the combination of ML inference and our Alignment Matrix algorithm demonstrated in this work. Attaining such a system is important for information storage because it would allow for real time filtering of information that has already been obtained, and thus better optimizes the overall storage system's sequencing costs by avoiding sequencing resource allocation to duplicate data. While we do find that our algorithm's runtime is far from a throughput that enables real-time application, increasing the overall time by increasing inference time is unlikely an approach to take to meet this system goal.

F. Assessment of Alignment Matrix Algorithm on Synthetic Nanopore Signals

While we have demonstrated that the `Alignment Matrix` algorithm provides average byte error rates that are competitive with the baseline tree-search and `Beam Trellis` algorithms, our analysis is limited to just a few unique encodings for each design due to the cost of synthesizing long DNA sequences (474 USD for our 2297 bp length strands). Thus, a challenge in providing an accurate value for the average byte error rate that we may extract from a data set of a large number of unique strands is being able to scale the analysis we have done to larger encoded strand sets. Accomplishing this through experimental means of synthesizing larger sets of unique oligos is not cost effective at this time. To this end, we consider analyzing open source tools aimed at allowing for *in silico* evaluation of basecaller algorithms based on simulated nanopore electrical signals.

We choose two *in silico* simulators that produce an electrical signal based on an input base sequence. One is *Squigulator* Gamaarachchi et al. (2023), a simulator that is based on using nominal descriptions of a nanopore device and applying Gaussian noise to these values to represent the stochastic nature of nanopore sequencing. The two main parameters of nanopore decoding are the current level that is realized for a set of bases, and the *dwell time* which describes the amount of time that some number of bases stay resident within the pore. The current level can be thought of as a unique identifier of a base sequence, while dwell time represents the amount of time that a current signal will be repeated. The nominal values that *Squigulator* uses for current levels are values that are publicly published by Oxford Nanopore Technologies for 5-mers (base sequence of length 5). A nominal value of dwell time is taken as the expected translocation speed of the molecule (450 bp/s assumed by Gamaarachchi et al. (2023)), and a number of samples that repeat the current signal of a given 5-mer can be derived from a sample of the Gaussian noise dwell

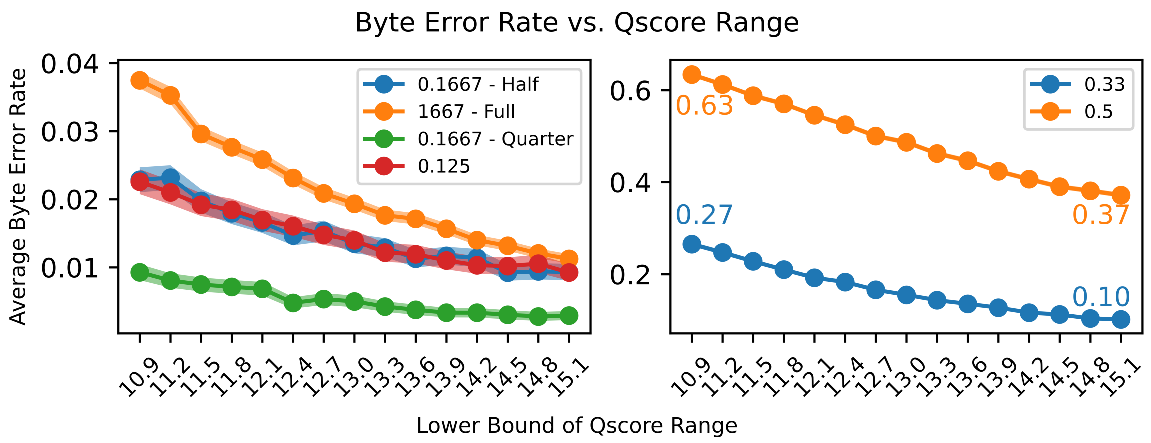

Fig. 16: Average byte error rate across all strands for each HEDGES design across a variety of Q score range bins, each with a range of 0.3. Error bands included for each line are calculated for a 95% confidence interval of Supplemental Equation 6. Legend entries represent different the different designs ($\phi_{\text{HEDGES}} = 0.1667 = 1/6$), and Quarter, Half, Full, represent the three lengths encoded used for $\phi_{\text{HEDGES}} = 1/6$ in the order of increasing length.

time and the rate at which the current is sampled (4 kHz assumed by Gamaarachchi et al. (2023)).

Another electrical signal simulator we consider is *DeepSimulator* Li et al. (2018). The insight of this model is that 5-mer models like those of Oxford Nanopore Technologies (`https://github.com/nanoporetech/kmer_models`) and *Squigulator* do not consider current variation that may be systematically created by the position of a 5-mer. That is, the current signal for a 5-mer is not just influenced by the 5-mer sequence itself, but also neighboring 5-mers. This context dependent model is implemented through the use of deep neural networks (DNN) to map base sequences to electrical signals while taking care to take into account time warping that can occur in the electrical signal. This process describes just the nominal modeled electrical signal. *DeepSimulator* also determines an estimation from experimental data on how long each signal should be sampled for to determine the amount of time that an electrical value for a 5-mer should repeat. They also include random noise on the extended electrical signal to provide a final simulated signal. The downside of this approach is that producing electrical signals is compute intensive. As reported by Gamaarachchi *et al.*, *Squigulator* can generate reads 3000x faster than *DeepSimulator*.

In our experiments evaluating these models we choose the *R9.4.1*-based models for each, as this is the pore version used in our nanopore sequencing runs. We choose 3 encodings for simulation. One being of the 0.167 HEDGES rate, and we simulate the entire strand design which includes buffers and poly-A regions (2297 bp). This design is simulated for 25 strands which make up the set in which the 7 strand experimental library was derived from. The other designs we study are HEDGES 0.333 (2249 bp) and 0.5 (2233 bp) where we adjust the number of bytes so that strand length stays close to constant. These strands encode the same information as HEDGES 0.167. Note, however, these HEDGES 0.333 and 0.5 rates do not encode the same data that was used for the *in vitro* nanopore analysis.

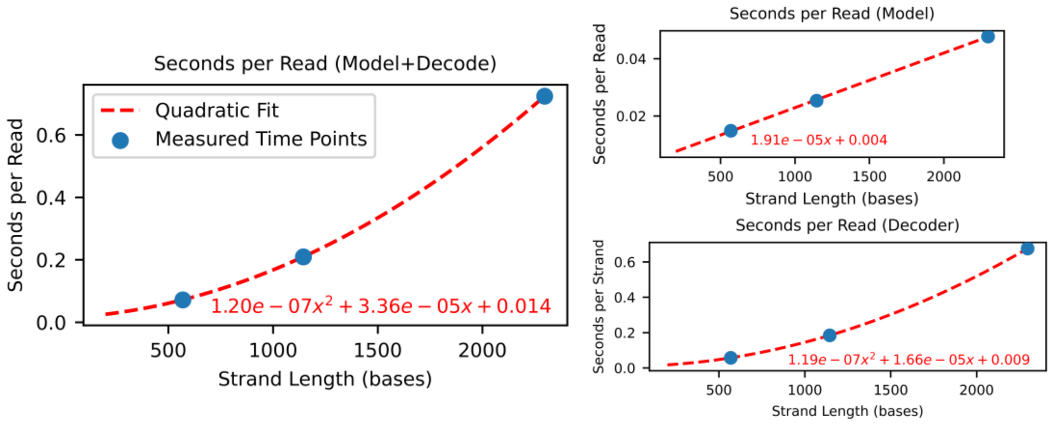

Fig. 17: Break down of time spent per read on average during the decoding process. **left:** Average seconds per read for the entire decoding process including the time taken to generate the CTC scores from the machine learning model (top right) and the time spent in the `Alignment Matrix` algorithm (bottom right).

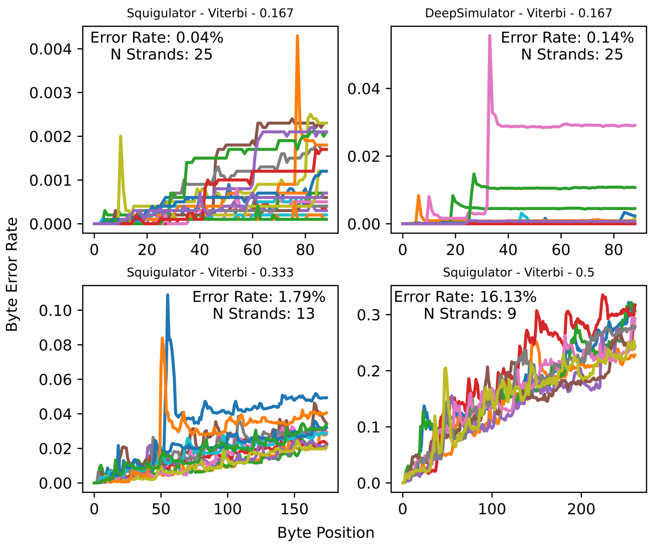

Fig. 18: Simulation of byte error rate through the use of 2 *in silico* nanopore electrical signal simulators. *DeepSimulator* is a machine learning model approach that attempts to take into account context of co-located bases to enhance simulator accuracy, *Squigulator* is a probabilistic approach based on Gaussian noise models. Every byte error rate for each encoded strand is taken from a sample of 9-10k simulated reads.

Figure 18 reports the byte error rates obtained for each of the evaluated designs and simulators when decoding with the `Alignment Matrix` algorithm. The first row of plots compares the two simulators for the same 0.167 HEDGES encoding. We have found that on average *DeepSimulator* has a higher average error rate across the 25 simulated strands at about 0.14% compared to just 0.04% for *Squigulator*. Furthermore, *DeepSimulator* has a population of strands that appear to systematically generate much higher error rates than others. A large peak in error rate at specific locations, with a flattening of the error rate afterwards indicates that there are specific base sequences that have a much higher probability of generating errors when interpreting the CTC matrix compared to other sequences in the strand. This may point to encodings that should be avoided, but it has been reported in previous work by Hamoum *et al.* Hamoum et al. (2021) and by Gamaarachchi *et al.* Gamaarachchi et al. (2023) that *DeepSimulator* tends to inject systematic errors into the basecall, an effect that is not characteristic of basecalls from experimental nanopore sequencing runs Hamoum et al. (2021).

Compared to the experimental error rates observed for 0.167 HEDGES in Table, both simulators under report the average error rate by two and 1 order of magnitude for *Squigulator* and *DeepSimulator* respectively. Furthermore, neither simulators have the characteristic increase in byte error rate across strand position that has been measured for each strand in the nanopore sequencing data set. Given the slower performance of *DeepSimualtor* and the already known issues of its error profile, we opted to simulate the remaining higher HEDGES rates only with *Squigulator*. Again, as the bottom row of Figure 18 illustrates, byte error rates are substantially smaller than what is measured experimentally. Experimentally we measure error rates of 22.55% and 56.34% for 0.333 and 0.5 HEDGES respectfully, but *Squigulator* reports just 1.79% and 16.13% for these encodings. We do note at these encoding rates, the expected positional increase in byte error rate is observed. However, we conclude that the two evaluated *in silico* nanopore simulators here do not accurately capture the CTC output generated by real nanopore reads. Furthermore, these observations motivate the study of a wider suite of *in silico* nanopore simulators within the context of information storage systems, especially those storage systems that utilize basecaller model probabilities such as ours.

G. Description of the Alignment Matrix Algorithm

Here we present pseudo code algorithmic descriptions of our `Alignment Matrix` algorithm that decodes a convolutional code's trellis by using CTC scores provided by some machine learning model. Our algorithm relies on manipulating values stored in multi-dimensional arrays. In our notation, we use brackets and indexes to represent retrieving a value from an array. For example, given a three-dimensional array A, we access a value at a row r, column c, and 3-rd dimension z by the statement $A[r, c, z]$. In scenarios when convenient, we utilize array slicing notation. For example, to generate a 1-dimensional column vector for the same three-dimensional array we write $A[:, c, z]$ where ":" represents retrieving all row values for a constant column c and 3rd dimension z. We also utilize multi-dimensional argmax functions that apply in specified dimension of an input array. For example, $B = \textbf{argmax}(A, \textbf{dim} = 2)$ outputs a 2-dimensional array with values of the positions of maximum values along the 3rd dimension of A. That is, $B[r, c] = \text{argmax}_i(A[r, c, i])$.

We break our complete algorithm down into four main sub-algorithm components. The first component is presented in Algorithm 3, which is the top level function to our custom base calling process. Key arguments for this function are the CTC scores ($\mathcal{C}$) that are generated by the machine learning model applied to the raw electrical signal produced by pores, and an array which describes the structure of the convolutional code's trellis ($\mathcal{I}$). The trellis structure array, $\mathcal{I}$, is 2-dimensional with each row representing a single state in the trellis and each column representing an edge entering each of these states. Each value stored in $\mathcal{I}$ is a tuple (i, v) which represents a predecessor state i with transition value v. The main responsibility of this function is to create arrays used to store alignment scoring, and to update those arrays after each step of the trellis. Initially, arrays are created from the viewpoint of transitioning away from each state. That is, we determine the symbols and scores for the edges that transition between step $l - 1$ and l of the trellis. Here, we assume that bases are generated by a generic convolutional encoding algorithm (*ConvolutionaGetNextBase*) that generates a base for some convolutional state h and transitional value e. Outgoing scores from each state are generated by the function *ForwardStep*, which is the core scoring mechanism of our novel algorithm. Then, we rearrange this information via $\mathcal{I}$ to create an incoming viewpoint for each state at step l such that we can determine the best incoming edge scores and their corresponding bases. Once the best incoming edges are determined, a 2-dimensional array representing the back trace path through the trellis is updated for each state at trellis step l. Once all $\mathcal{L}$ steps of the trellis complete, *StringFromBacktarce* is used to generate the final basecall from the back trace array.

The scoring function, *ForwardStep* determines scores in 2 phases. The first phase is given in Algorithm 1. Here, an array

representing the newly added bases (and inserted blanks) along each state's outgoing paths, B_{targets}, is used to determine where symbols repeat. For example, for a given state h, transition e, and base G on edge e leaving h the vector $B_{\text{targets}}[h, e, :]$ represents $b' - G$. Here, b' is the most recent base that has been aligned for the message currently stored at h. Thus, $\mathcal{F}[:, h]$ is precisely the alignment information stored for base b'. Knowing where repeats occur is important, as there cannot be any alignment that transitions between the matching bases without passing through a blank symbol. Based on this, we create a masking array M which is used to mask out illegal transition scores during the core alignment calculations. Subsequently, all necessary arrays are transferred from CPU memory to a *GPU* device's memory and threading parameters are constructed for parallel execution of the *ForwardStepKernel* function. We assume *CUDA*-style notation for parameterizing the organization of threads and local (shared) memory. That is, threads are organized in 3-dimensional blocks, and blocks are organized on a 3-dimensional grid. In our algorithm we allocate shared memory for an array $\mathcal{A}$ which is designed to hold intermediate alignment scores of single time steps for all symbols whose alignment requires calculation. Finally, *GPU.LaunchKernel()* is performed to start execution of the kernel on a GPU device.

The core calculations that determine scores of bases that lie along each edge leaving a trellis state are performed in Algorithm 2. To begin, the array $\mathcal{A}$ is initialized with values that represent the alignment of symbols at time $T_L - 1$, e.g. the time step previous to the lowest time allowed. The array $\mathcal{A}$ has a value reserved for each base in B_{targets} (plus 1 padding base) for the trellis states and edges that execute in the same thread block. We also include a fourth dimension to $\mathcal{A}$ of length 2 to enable writing the next values of $\mathcal{A}$ without overwriting the current values that may still be needed by other threads. As the main loop of this algorithm proceeds, 3 values are read from $\mathcal{A}$ for each symbol that requires alignment calculation. The 3 values represent alignment scores of the previous time step coming from the same symbol, the 1st previous symbol, or the symbol 2 positions away. These values are combined with the raw CTC score for a base b at time t ($\mathcal{C}[t, b]$) to general a final score S_{final}. S_{final} is then used to fill the $\mathcal{A}$ array position of each base, and a rolling accumulation across time steps of final scores, R, is kept for each base. At the end of each loop iteration, the last base scores are written to $\mathcal{F}_{out}$, and threads in a block are synchronized so that updated $\mathcal{A}$ values are visible to every thread in the subsequent iteration. As the algorithm terminates, the rolling score R is written to S_{out} in the thread that represents the final base.

After repeating the process $\mathcal{L}$ times of calculating scores and choosing the best incoming edges for each trellis state, Algorithm 4 is executed to build a final base call from chosen paths. For this algorithm, a start state is chosen based on the position of the best score in S_{current} of Algorithm 3. Starting at this state, and the last position in the message, the BT_{index} array is traversed based on the index stored for each state that points to the best state at the previous position in the message. Along the way, the corresponding positions in BT_{bases} are retrieved to build the output basecall. After building a complete message, the string is returned and our algorithm finishes.

Algorithm 1: Set up function for launching the core GPU kernel threads that calculate alignments in parallel.

Input: A $\mathcal{T} \times 5$ array of CTC log-probability scores $\mathcal{C}$, a $\mathcal{T} \times \mathcal{H}$ array of alignment scores $\mathcal{F}$ representing log-probabilities, a $\mathcal{H} \times \mathcal{E} \times 2$ array of outgoing strings for each edge from each state B_{trans}, an integer representing the message index l, an $\mathcal{H} \times \mathcal{E}$ array of integers representing the start base for each new path from each state B_{start}, the message length $\mathcal{L}$

Output: A $\mathcal{H} \times \mathcal{E}$ array of edge scores for each state S_{out}, a $\mathcal{T} \times \mathcal{H} \times \mathcal{E}$ array of alignment scores $\mathcal{F}_{\text{out}}$

```
Function ForwardStep(C, B_start, B_trans, F, l, L):
    T_L ← l
    T_U ← T − L + l + 1
    B_targets ← H × E × 3 array initialized to 0   ▷ Concatenate start base of each state with transition strings.
    for h in {0, ..., H − 1} do
        for e in {0, ..., E − 1} do
            for k in {0, ..., 2} do
                p ← k
                if B_start ≠ ∅ then p ← k − 1
                if B_start == ∅ or k¿0 then B_targets[h, e, k] ← B_trans[h, e, p − 1]
                else B_targets[h, e, 0] ← B_start[h, e]
            end
        end
    end
    L ← size(B_trans)[2]                                    ▷ Get size of 3rd dimension
    M ← H × E × L array initialized to −∞                   ▷ Mask repeat symbols
    if B_start ≠ ∅ then
        for h in {0, ..., H − 1} do
            for e in {0, ..., E − 1} do
                for i in {2, ..., L} do
                    if B_targets[h, e, i − 2] ≠ B_targets[h, e, i] then M[h, e, i − 1] ← 0
                end
            end
        end
    end
    S_out ← H × E array initialized with 0
    F_out ← T × H × E array initialized to −∞
    move F, C, M, S_out, F_out, B_targets to GPU
    T_block ← 128                                           ▷ Number of threads per block
    H_per-block ← ⌊T_block/(L · E)⌋                         ▷ Trellis states per thread block
    H_blocks ← ⌊H/H_per-block⌋ + 1                          ▷ Total blocks on kernel launch
    A ← H_per-block × E × (L + 2) × 2 array initialized to 0
    ▷ Kernel launch for parallel GPU alignments
    GPU.LaunchKernel(grid=(H_blocks,1,1), block=(L, H_per-block, E), shared_mem = A, args= ( C, F, M, B_targets, S_out, F_out, T_L,
     T_U), kernel=ForwardStepKernel)
    return S_out, F_out
End Function
```

Algorithm 2: GPU kernel algorithm used to perform the core alignment calculations.

Input: A $\mathcal{T} \times 5$ array of CTC log-probability scores $\mathcal{C}$, a $\mathcal{T} \times \mathcal{H}$ array of alignment scores $\mathcal{F}$ representing log-probabilities, a $\mathcal{H} \times \mathcal{E} \times L$ masking array M, a $\mathcal{H} \times \mathcal{E} \times j$ array storing the bases to be aligned to the CTC matrix B_{targets}, a $\mathcal{H} \times \mathcal{E}$ array used to store output calculations that score edges leaving each trellis state S_{out}, a $\mathcal{T} \times \mathcal{H} \times \mathcal{E}$ array used to store alignment scores across all CTC times for each edge leaving each trellis state $\mathcal{F}_{\text{out}}$, the lower time limit T_L to align to in the CTC matrix, the upper time limit T_U to align to in the CTC matrix.

Kernel Parameters: An array $\mathcal{A}$ stored in shared memory with dimensions $\mathcal{H} \times \mathcal{E} \times (L+2) \times 2$ storing intermediate alignments of symbols, a 3-tuple of integers (B_x, B_y, B_z) providing the block index of a thread, a 3-tuple of integers $(B_{d,x}, B_{d,y}, B_{d,z})$ providing the dimensions of each block in each dimension, a 3-tuple of integers (T_x, T_y, T_z) providing the thread index of a thread within a block.

Function ForwardStepKernel($\mathcal{C}$, $\mathcal{F}$, M, $B_{targets}$, S_{out}, $\mathcal{F}_{out}$, T_L, T_U):

- ▷ Each thread on a GPU device executes an instance of this algorithm.
- $L_{\text{idx}} \leftarrow T_x,\ E_{\text{idx}} \leftarrow T_z,\ H_{\text{idx}} \leftarrow T_y + B_x \cdot B_{d,y},\ BH_{\text{idx}} \leftarrow T_y$
- $T_{\text{mod}} \leftarrow T_L \mod 2$ ▷ Tracks which sub-array of $\mathcal{A}$ to fill.
- ▷ Initialize shared memory to set up dynamic programming loop.
- **if** $L_{idx} == 0$ **then** $\mathcal{A}[BH_{\text{idx}}, E_{\text{idx}}, L_{\text{idx}}, T_{\text{mod}}] \leftarrow -\infty$
- **else if** $L_{idx} == 1$ **then**
  - $\mathcal{F}_v \leftarrow -\infty$
  - **if** $T_L \neq 0$ **then** $F_v \leftarrow \mathcal{F}[T_L - 1, H_{\text{idx}}]$;
  - $\mathcal{A}[BH_{\text{idx}}, E_{\text{idx}}, L_{\text{idx}}, T_{\text{mod}}] \leftarrow \mathcal{F}_v$
- **else**
  - $\mathcal{A}_v \leftarrow 0$ ▷ Handle case of first base.
  - **if** $T_L \neq 0$ **then** $A_v \leftarrow -\infty$
  - $\mathcal{A}[BH_{\text{idx}}, E_{\text{idx}}, L_{\text{idx}} + 2, T_{\text{mod}}] \leftarrow \mathcal{A}_v$
- $M_v \leftarrow M[H_{\text{idx}}, E_{\text{idx}}, L_{\text{idx}}]$ ▷ Get mask value for thread.
- $b \leftarrow B_{\text{targets}}[H_{\text{idx}}, E_{\text{idx}}, L_{\text{idx}} + 1]$ ▷ Get target base for thread.
- $R \leftarrow -\infty$ ▷ Rolling reduction value.
- **for** t *in* $\{T_L, ..., T_U\}$ **do**
  - $T_{\text{next-mod}} \leftarrow T_{\text{mod}} + 1 \mod 2$
  - ▷ Determine score for time t based on results of $t-1$ placed in $\mathcal{A}$
  - $a \leftarrow \mathcal{A}[BH_{\text{idx}}, E_{\text{idx}}, L_{\text{idx}} + 2, T_{\text{mod}}]$
  - $a_1 \leftarrow \mathcal{A}[BH_{\text{idx}}, E_{\text{idx}}, L_{\text{idx}} + 2 - 1, T_{\text{mod}}]$
  - $a_2 \leftarrow \mathcal{A}[BH_{\text{idx}}, E_{\text{idx}}, L_{\text{idx}} + 2 - 2, T_{\text{mod}}] + M_v$
  - $S_{\text{final}} \leftarrow \mathcal{C}[t, b] + \log(e^{a} + e^{a_1} + e^{a_2})$ ▷ Final score for symbol at t.
  - ▷ Update $\mathcal{A}$ to enable score calculations at $t+1$.
  - $\mathcal{A}[BH_{\text{idx}}, E_{\text{idx}}, L_{\text{idx}} + 2, T_{\text{next-mod}}] \leftarrow S_{\text{final}}$
  - **if** $L_{idx} == 0$ **then** $\mathcal{A}[BH_{\text{idx}}, E_{\text{idx}}, L_{\text{idx}}, T_{\text{next-mod}}] \leftarrow -\infty$
  - **else if** $L_{idx} == 1$ **then** $\mathcal{A}[BH_{\text{idx}}, E_{\text{idx}}, L_{\text{idx}}, T_{\text{next-mod}}] \leftarrow \mathcal{F}[t, H_{\text{idx}}]$
  - GPU.SyncBlock() ▷ Ensure synchronization of dependent symbols in parallel threads.
  - ▷ Write alignment for final base at time t
  - **if** $L_{idx} == L - 1$ **then** $\mathcal{F}_{\text{out}}[t, H_{\text{idx}}, E_{\text{idx}}] \leftarrow S_{\text{final}}$
  - $S_{\text{next}} \leftarrow 0$
  - **if** $t + 1 \leq T_U$ **then** $S_{\text{next}} \leftarrow \mathcal{C}[t+1, b]$
  - $R \leftarrow \log(e^{R} + e^{S_{\text{final}} + \log(1 - e^{S_{\text{next}}})})$
  - $T_{\text{mod}} \leftarrow T_{\text{next-mod}}$
- **end**
- ▷ Write out aggregate scores.
- **if** $L_{idx} == L - 1$ **then** $S_{\text{out}}[H_{\text{idx}}, E_{\text{idx}}] \leftarrow R$

End Function

Algorithm 3: Main basecalling function that determines a sequence of bases from an input trellis structure and CTC scoring of bases across a read's electrical signal.

```
Input: Integer number of trellis states H, Integer number of transitions per trellis state E, a H × E array of tuples I where each
       element of I is a tuple (i, v) where i represents an incoming trellis state and v represents the value on the transition,
       integer of number of bases in message L, a T × 5 array of CTC log-probability scores C
Output: A basecalled sequence of bases S
Function BasecallFromCTC(E,H,I,L,C):
    BT_index ← H × L array initiated to 0                          ▷ Indexes pointing to back trace paths
    BT_bases ← H × L array initiated to 0                          ▷ Bases to added on back trace
    F ← T × H array initiated to −∞                                ▷ Tracking each state's current alignment
    B_trans ← H × E × 2 array initiated to 0
    S_current ← H-length array initiated to −∞
    for l in {0...L − 1} do
        for h in {0...H − 1} do
            for e in {0...E − 1} do
                b ← ConvolutionalGetNextBase(h, e)                 ▷ Generate guess for next base.
                B_trans[h, e, 1] ← b
            end
        end
        B_start ← ∅
        ▷ Get the previous added base.
        if l > 0 then  B_start ← BT_bases[:, l − 1]
        S_out, F_out ← ForwardStep(C, B_start, B_trans, F, l, L)
        for h in {0, ..., H − 1} do
            for e in {0, ..., E − 1} do
                ▷ Re-arrange scores so each row (state) is filled with incoming scores.
                S_states[h, e] ← S_out[I[h, e].i, I[h, e].v]
            end
        end
        E_max ← argmax(S_states, dim = 1)                          ▷ Locations of best edge for each state.
        for h in {0...H − 1} do
            ▷ Update back trace and alignment for trellis states
            S_current[h] ← S_states[h, E_max[h]]
            h_best ← I[h, E_max[h]].i
            v_best ← I[h, E_max[h]].v
            BT_index[h, l] ← I[h, E_max[h]].i
            BT_bases[h, l] ← B_trans[h_best, v_best, 1]
            for t in {0, ..., T − 1} do  F[t, h] ← F_out[t, h_best, v_best]
        end
    end
    State_start ← argmax_i(S_current[i])
    return StringFromBacktrace(BT_index, BT_bases, State_start)
End Function
```

Algorithm 4: Algorithm that derives an output base sequence based on the computed backtrace matrices.

```
Input: A H × L array of integers BT_index, A H × L array of integers BT_bases, an integer h_start
Output: A basecalled sequence of bases S
Function StringFromBacktrace(BT_index,BT_bases,h_start):
    h_current ← h_start
    B_map ← ["−", "A", "C", "G", "T"]                              ▷ Map an integer to a base character.
    S ← ""                                                         ▷ Initialize return string to be empty
    for i in {0, L − 1} do
        S ← S + B_map[BT_bases[h_current, i]]
        if i==0 then  break
        h_current ← BT_index[h_current, i]
    end
    return reverse(S)                                              ▷ Return reverse of built strand from back trace.
End Function
```

Table 1. Sequencing run and basecaller specification for the nanopore sequencing of each encoded strand. The designs labeled with F (full)/H (half)/Q (quarter) refer to the three sizes of the $1/6$ HEDGES rates code that was synthesized in decreasing length order.

Design	Molecule	Index	Sequencing Type	Flowcell	Flowcell ID	minkow version	basecaller	basecaller version	basecall model
$\frac{1}{6}-$F	rna	1	single	FLO-MIN106	FAU30563	22.05.7	guppy	6.1.5	rna_r9.4.1_70bps_hac
$\frac{1}{6}-$F	rna	2	single	FLO-MIN106	FAU49457	22.05.7	guppy	6.1.5	rna_r9.4.1_70bps_hac
$\frac{1}{6}-$F	dna	1	single	FLO-MIN106	FAV18701	22.10.5	guppy	6.3.8	2021-05-17_dna_r9.4.1_ minion_384_d37a2ab9
$\frac{1}{6}-$F	dna	2	single	FLO-MIN106	FAV18737	22.10.5	guppy	6.3.8	2021-05-17_dna_r9.4.1_minion_384_d37a2ab9
$\frac{1}{6}-$F	dna	14-18	pooled	FLO-MIN106	FAW81322	23.07.12	dorado	7.1.4	dna_r9.4.1_e8_hac@v3.3
$\frac{1}{6}$ -Q	dna	3,4	pooled	FLO-MIN106	FAW81553	23.07.12	dorado	7.1.4	dna_r9.4.1_e8_hac@v3.3
$\frac{1}{2}$	dna	1,2	pooled	FLO-MIN106	FAW81799	23.07.12	dorado	7.1.4	dna_r9.4.1_e8_hac@v3.3
$\frac{1}{3}$	dna	1,2	pooled	FLO-MIN106	FAW81801	23.07.12	dorado	7.1.4	dna_r9.4.1_e8_hac@v3.3
$\frac{1}{6}-$H	dna	1,2	pooled	FLO-MIN106	FAW81721	23.07.12	dorado	7.1.4	dna_r9.4.1_e8_hac@v3.3
$\frac{1}{8}$	dna	1,2	pooled	FLO-MIN106	FAX97481	23.07.12	dorado	7.1.4	dna_r9.4.1_e8_hac@v3.3
